# Estimating Dose Painting Effects in Radiotherapy: A Mathematical Model

**DOI:** 10.1371/journal.pone.0089380

**Published:** 2014-02-26

**Authors:** Juan Carlos López Alfonso, Nick Jagiella, Luis Núñez, Miguel A. Herrero, Dirk Drasdo

**Affiliations:** 1 Department of Applied Mathematics, Faculty of Mathematics, Universidad Complutense de Madrid, Madrid, Spain; 2 Institut National de Recherche en Informatique et en Automatique (INRIA), Domaine de Voluceau - Rocquencourt, Paris, France; 3 Institute of Computational Biology, Helmholtz Center Munich, German Research Center for Environmental Health, Neuherberg, Germany; 4 Radiophysics Department, Hospital Universitario Puerta de Hierro, Majadahonda, Madrid, Spain; 5 University of Paris 6 (UPMC), CNRS UMR 7598, Laboratoire Jacques-Louis Lions, Paris, France; 6 Interdisciplinary Center for Bioinformatics (IZBI), University of Leipzig, Leipzig, Germany; Fondazione Edmund Mach, Research and Innovation Centre, Italy

## Abstract

Tumor heterogeneity is widely considered to be a determinant factor in tumor progression and in particular in its recurrence after therapy. Unfortunately, current medical techniques are unable to deduce clinically relevant information about tumor heterogeneity by means of non-invasive methods. As a consequence, when radiotherapy is used as a treatment of choice, radiation dosimetries are prescribed under the assumption that the malignancy targeted is of a homogeneous nature. In this work we discuss the effects of different radiation dose distributions on heterogeneous tumors by means of an individual cell-based model. To that end, a case is considered where two tumor cell phenotypes are present, which we assume to strongly differ in their respective cell cycle duration and radiosensitivity properties. We show herein that, as a result of such differences, the spatial distribution of the corresponding phenotypes, whence the resulting tumor heterogeneity can be predicted as growth proceeds. In particular, we show that if we start from a situation where a majority of ordinary cancer cells (CCs) and a minority of cancer stem cells (CSCs) are randomly distributed, and we assume that the length of CSC cycle is significantly longer than that of CCs, then CSCs become concentrated at an inner region as tumor grows. As a consequence we obtain that if CSCs are assumed to be more resistant to radiation than CCs, heterogeneous dosimetries can be selected to enhance tumor control by boosting radiation in the region occupied by the more radioresistant tumor cell phenotype. It is also shown that, when compared with homogeneous dose distributions as those being currently delivered in clinical practice, such heterogeneous radiation dosimetries fare always better than their homogeneous counterparts. Finally, limitations to our assumptions and their resulting clinical implications will be discussed.

## Introduction

Radiotherapy, the use of ionizing radiation to eliminate pathological tissues, is a treatment of choice for more than 50% of cancer patients diagnosed with solid tumors [1]. Technical and methodological advances have allowed radiation oncology to achieve local tumor control in a considerable number of patients. However, locoregional recurrence (LRR) remains a problem in many clinical settings. For example, a recent study in patients with Stage III lung cancer found a 5-year LRR rate of 31% [2]. In Glioblastoma Multiforme (GBM), the most common and aggressive malignant primary brain tumor, LRR approaches 90% [3]. In such critical cases, radiotherapy usually results in an initial shrinkage of malignancies, followed by a subsequent growth recovery that cannot be checked even by resorting to larger radiation doses.

The onset of radioresistance, and its resulting poor prognosis, is strongly correlated with the development of significant intratumoral heterogeneity. For that reason, there is growing interest in the clinical significance of tumor heterogeneity. In different works have been recently demonstrated extensive genetic variations in tumor cells due to intratumoral evolution [4], [5]. Moreover, tissue-level heterogeneity due to variations in vascular density and blood flow has been long since evident in clinical medical imaging. In recent years, accumulating evidence suggests that tumor heterogeneity is a key factor in the development of therapeutic resistance and therefore in radiation therapy outcomes [6], [7], [8]. As a consequence, increasing attention is being paid to “dose painting” (or “dose sculpting”), a technique which consists in prescribing different radiation dosimetries to different regions within a given tumor, so that irradiation be boosted in more radioresistant (for instance, hypoxic, quiescent, etc.) regions [9], [10]. This strategy, which is in sharp contrast with the still prevailing homogeneous radiation dose delivery approach recommended by International Commission on Radiation Units (ICRU) reports (50, 1993; 62, 1999 and 83, 2010; see [11], [12] and [13] respectively), has been made possible by the availability of high-precision clinical particle accelerators, and looks particularly promising in those cases where current treatment techniques fail to provide sufficient tumor control.

However, in order for dose painting to show its full power, detailed information is needed about the internal structure of the tumor to be irradiated. Such information should ideally be provided by medical imaging techniques. These however are not yet able to distinguish different radiosensitivity regions except in a few cases, commonly related to hypoxia effects. On the other hand, even a modest miscalculation in the sizes of different radiosensitivity subvolumes has been suggested to produce serious consequences in clinical outcomes [14]. In view of current technical limitations, the question thus arises of providing tools to *a)* obtain as much information as possible about tumor heterogeneity before a radiation dosimetry plan is prescribed, *b)* simulate the effects of dose painting therapies which take into account whatever heterogeneity data are available, and *c)* compare such simulations with those corresponding to standard homogeneous radiation dose distributions currently delivered in clinical practice.

The work herein reported intends to yield some insight into these issues. More precisely, in the sequel a mathematical model for heterogeneous tumor growth is formulated, and the effects of various radiation dose distributions on it are investigated by means of computer simulations. Specifically, we consider a situation where two tumor cell phenotypes, cancer cell (CC) and cancer stem cell (CSC), are present at an early stage, when the tumor consists of about 10^5^ cells in total. Concerning CCs and CSCs, we have assumed that *i)* CSCs represent only a small percentage of the total number of cells at that stage (say, about 15%), *ii)* CSCs have a significantly longer cell cycle duration than CCs and can replicate indefinitely, while CCs can perform only a limited number of cell divisions, and *iii)* CCs and CSCs show quite different resistance to radiation, CSCs being more radioresistant than CCs. These biological and radiobiological features have been reported in the literature, specifically for Glioblastoma Multiforme (GBM), where there is mounting evidence of CSCs presence in GBM tumors (cf. for instance [8], [15], [16], [17]). Growth of the heterogeneous tumor thus resulting is simulated by means of an agent-based model in which each cell is individually represented [18], [19]. Tumor growth is kept track of until a size of approximately 10^6^ cells is attained, which roughly corresponds to a spheroid of about 1 cubic millimeter in size, a typical volume in multi-cellular spheroids (MCS) *in vitro* growth. At that stage, different (homogeneous and heterogeneous) radiation dose distributions are simulated using the Linear-Quadratic (LQ) model [20], [21], and their effects compared.

An interesting consequence of *i)* and *ii)* above is then shown to be that, as tumor grows, most of the CSCs concentrate themselves within the tumor core, irrespective of their initial distribution at an earlier stage. This fact, which will be described to be inversely correlated with cell migration rates when migration is not inhibited by cell-cell adhesion (which is the case, for instance, after cells undergo an epithelial-mesenchymal transition (EMT) [22]), is then used together with *iii)* to simulate the effects of different radiation dosimetries to achieve tumor control, or in the case where this cannot be obtained, to compare tumor heterogeneity (seen as an indicator of malignancy in terms of the proportion of CSCs) before and after treatment has been delivered. In this context it will be shown that for a given amount of radiation, heterogeneous dose distributions, where different radiation doses are delivered at different regions of the tumor according to the presence of more radioresistant cells there, invariably fare better than homogeneous ones when sufficient information about tumor spatial heterogeneity is available. In our case, such information will be shown to follow from assumptions *i)* and *ii)* above. It should be noticed that hypotheses *i)*, *ii)* and *iii)* are amenable to experimental validation, at least *in vitro*.

Our work can be considered as a preliminary step towards analyzing preclinical models where larger tumors (of the order of cubic centimeters) should be dealt with, several tumor cell phenotypes would simultaneously be present (possibly as a consequence of mutations) as tumor expands, and vascular networks, immune response, and hypoxic and necrotic effects are also taken into account. While the case herein considered is still far from that situation, the simplicity of the setting selected allows us to stress the consequences derived from the minimal number of biological and radiobiological assumptions made on the tumor cell phenotypes involved. This last is particularly relevant in view of the scarcity of *in vivo* biological parameter measures available. Scaling results up to larger tumor sizes, as well as increasing phenotypic and anatomical complexity appear as feasible within the same approach, but only after key biological data retrievable by non-invasive probing had been identified, and their impact on tumor growth elucidated, an objective toward we intend to contribute with this work.

We conclude this introduction by observing that considerable attention is being currently paid to mathematical modeling as a tool towards designing patient-tailored and adaptive therapies; see for instance [22], [23], [24], [25], [26], [27], [28], [29], [30] and [31]. In particular, radiotherapy modeling and simulations have been addressed in [32], [33], [34], [35], [36], [37], [38], [39] and [40], as well as in [41], [42] and [43] where GBM cases are considered. Mathematical models and computer simulations on the impact of the presence of CSCs in tumor therapies have been discussed in [32], [44] and more recently in [43], where focus is made in a GBM case. It is worth to be stressed, however that in the cases previously mentioned, the total number of cells simulated (and thus the resulting structural complexity) remained way below that of the computer simulations arrived at in our current work.

## Materials and Methods

### Tumor Cell Phenotypes Assumptions

For definiteness model parameter values corresponding to Glioblastoma Multiforme (GBM) cell lines have been used [45], [46], [47]. More precisely, we consider a tumor where two different phenotypes coexist at an early stage, when we assume a preponderant (approximately 85% of the total tumor volume) proportion of a tumor cell phenotype denoted as CC (cancer cell) coexisting with a second tumor cell phenotype CSC (cancer stem cell), randomly distributed, that roughly represents 15% of the total population at that stage. Both phenotypes CC and CSC are supposed to possess markedly different biological and radiobiological properties. In particular, we assume:

**P1.-** The duration of cell cycle for CCs is significantly shorter than that of CSCs. In particular, CCs are assumed to divide every 26 hours. Then, for tumor cell phenotype CSC three cases are considered, corresponding respectively to a CSC cycle duration of 96 hours (four days), 72 hours (three days) and 48 hours (two days). Moreover, CCs are assumed to divide a maximum of 15 times, while CSCs are able to replicate indefinitely.

Concerning property P1, it is currently assumed that CSCs proliferate at a slower pace than ordinary cancer cells (see for instance [16], [48], [49], [50], [51], [52], [53], [54], [55] and [56]). Actually, as observed in the references previously quoted, slow-cycling is to be expected from CSCs since such cells belong to tumor phenotypes that are highly resistant to current therapies (radiotherapy, chemotherapy or combined) and these are targeted at killing cycling cells. On the other hand, recent *in vivo* experiments in a mouse model of Glioblastoma to identify and isolate CSCs through genetically engineered mice demonstrate the presence of a small pool of slow-cycling and highly tumorigenic cells that retain long-term self-renewal ability [55], [57]. We notice that cell cycle durations of 24 *h*–26 *h* for GBM have been reported [43], [45], [58], although considerably different cell cycle durations, which in particular include the values herein considered for CSCs, have been noticed as well [45], [59]. Concerning the assumption on the maximum number of CCs replications (see for instance [60]), we have selected the value 15 (cf. [32], [61], [62]), but our results continue to hold if this number is slightly changed. Actually, an arbitrary increase in CSC cycle duration is always compatible with our results, as long as CC cycle duration continues to be significantly faster.

In the course of tumor growth, each of the previous tumor cell phenotypes may transiently enter in a quiescent, non-proliferating stage, due to contact inhibition. Moreover, replication of CCs is always supposed to be symmetric. On the other hand, CSCs will be assumed to sustain either symmetric or asymmetric division, in which case one CSC and one CC will result from replication. Evidence for asymmetric division for CSCs, has been reported in [63], [64], [65]. Since reliable estimates about actual probabilities of asymmetric division 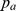 do not seem to be available as yet, computer simulations will be performed for different choices of that model parameter, namely 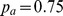, 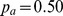 and 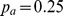 (cf. for instance [32], [61]).

A second key assumption is:

**P2.-** When irradiated, CSCs are significantly more resistant to radiation than CCs.

As a matter of fact, CSCs have been described as a comparatively small subpopulation that is highly radioresistant [15], [17], [61], [66]. Radioresistance and surviving cell fractions are estimated by means of the standard Linear-Quadratic (LQ) model [20], [21]. According to it, the surviving fraction of cells after a radiation dose 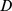 has been delivered, 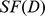, is given by:
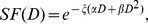
(1)where 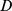 is usually measured in Grays (Gy) (1 *Gy* is 1 Joule per Kilogram), 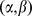 are the so-called radiosensitivity parameters, which depend of the cell phenotype considered, and 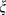 is a parameter introduced, as in [32], to distinguish the different radiosensitivities of the proliferating and quiescent states for CCs and CSCs. Actually, cells in a quiescent state (in the 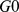 cell cycle phase) are known to be more resistant to radiation than their non-quiescent counterparts [67].

It should be noticed that, when estimating the impact of radiation according to the LQ model, what matters is the particular combination of 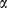 and 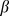 that appears in (1), which provides the surviving cell fractions, rather than the separate values of 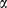 and 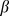 by themselves. For definiteness, we take in the sequel 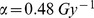, 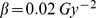 and 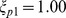 for proliferating CCs. These radiosensitivity parameters have been reported in [47], where *in vitro* estimates on surviving cell fractions at 2.0 *Gy*, 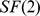, can be found for different GBM cell lines (see also [68]); similar values for 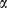 and 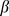 have been recently proposed in [43]. In particular, 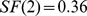 for proliferating CCs in our case (to be compared to the value 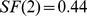 corresponding to 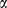 and 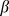 parameters proposed in [43]). For quiescent CCs we take 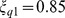, so that the corresponding value is 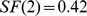. For proliferating CSCs the value 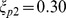 (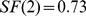) is taken, whereas for quiescent CSCs the value 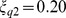 (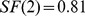) is selected. Also, such surviving cell fraction ranges at 2.0 *Gy* have been observed and reported in the literature (cf. [43], [68], [69]). We point out that the results to be described in this work continue to hold when the values selected for the radiosensitivity parameters 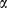 and 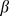 undergo considerable variations, which in particular include the ranges considered in the references quoted above. As a matter of fact, once assumptions P1 and P2 are made, our model is shown to be quite robust with respect to changes in its parameters.

### A Three-dimensional (3D) Model of Stochastic Tumor Growth

Different mathematical models of tumor growth and its radiation response have been reported in the literature. For instance, tumor growth models and radiation effects with continuous and discrete populations have been reviewed in [70], [71], [72] (see also [33], [34], [35], [36], [73], [74], [75], [76] and [77] for more details). On the other hand, the effects of different radiation dosimetries have been considered in [32], [33], [34], also in [43], [78] for fractionated radiotherapy and in [79] for a case of stereotactic radiosurgery. However, little seems to be known concerning mathematical modeling and computer simulations on the effects of heterogeneous radiation dose distributions on heterogeneous tumors, as in the case herein examined.

**Figure 1:**
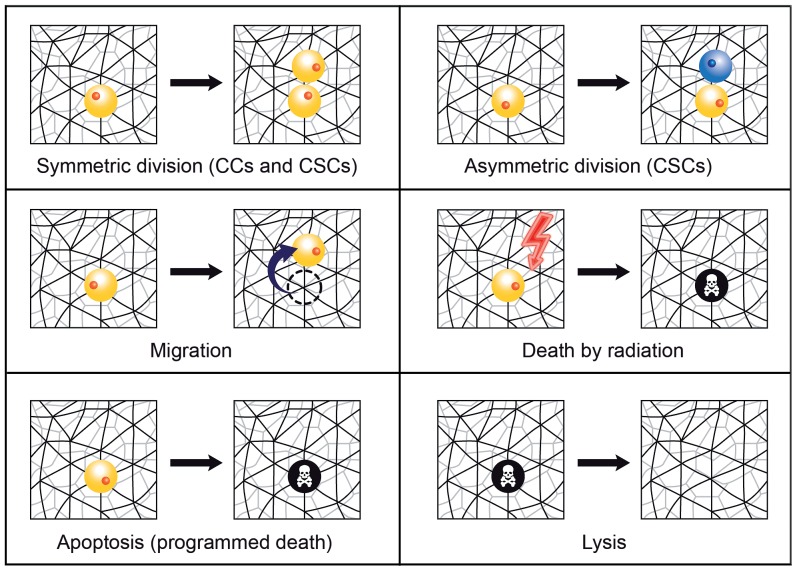
Cell processes mimicked in the model of tumor growth. Schematic representation of the cell processes considered in the model of tumor growth (symmetric and asymmetric division, migration, death by radiation, apoptosis (programmed death) and lysis (removal of debris)). Notice that CSCs can perform all these cell processes, while replication of CCs is always supposed to be symmetric. See Document S1 for further details.

**Figure 2:**
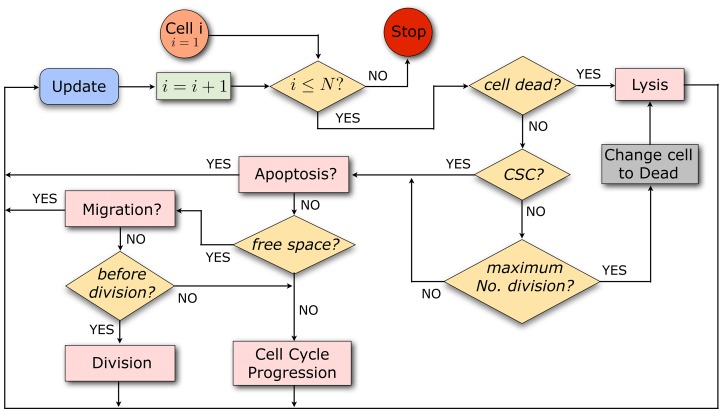
Scheme showing the possible actions that a cell is able to perform in the model. As long as the population size is below a prescribed maximum 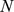, it is first tested whether a cell is dead. If so, it undergoes lysis at a certain rate. Alive cells are classified according to CSCs and CCs; CCs die and are subject to lysis with a certain rate once they have performed the maximum number of cell divisions prescribed. CCs not having yet reached the maximum number of cell divisions and CSCs can undergo apoptosis. Those cells that do not go through apoptosis can migrate if free space is available. If they do not migrate and have sufficiently advanced in the cell cycle, they divide. If those cells have not yet reached the end of G2-phase, then they continue to progress in the cell cycle. Cells with no free space available at neighboring sites can only progress in the cell cycle. Concerning radiation effects, cells are picked randomly and killed according to the corresponding surviving cell fraction estimate. See Document S1 for details on the technical implementation of the model algorithm.

The model of tumor growth implemented in this work is as follows. Within the growing tumor, both tumor cell phenotypes, CC and CSC, will be subject to the same kinetic rules. More precisely, following [18], [80], [81], a three-dimensional (3D) cellular automata (CA) model for tumor growth is developed, where each cell is considered as an individual agent. In particular, each cell (whether CC or CSC) occupies a single node in a 3D unstructured lattice (a lattice with no rotational or translational symmetry [81]) thus avoiding symmetry artifacts. Cell division, migration, apoptosis (programmed death) and lysis (removal of debris) have been included and are represented by stochastic processes. Accordingly, each kinetic rule is characterized by a rate, and the governing equation to be solved is a multivariate master equation (see equation (2)). Figures 1 and 2 show a sketch of the processes included and a scheme describing the possible actions that a cell is able to perform in the mathematical model respectively (for further details, see Document S1 provided). Nutrient-limited growth is not accounted for in our model. This issue, as well as others, could be included at the expense of increasing complexity by adding degrees of freedom in the computer simulations, but they are not expected to play a significant role in a tumor cell colony of the size considered, which may be assumed to be fully oxygenated [80]. At any rate, for tumors of the size considered in this work our assumption is not unlikely. For instance, *NIH3T3* cells form tumors of size larger than 1 *cm* ^3^ without apparent necrotic core even though micro-lesions may be observed [80].

As to the rules of the model of tumor growth, proliferation is only possible for cells located at a node having at least one free neighbor in the lattice. In the case that all neighbor sites are occupied, a cell enters in a quiescent state due to contact inhibition. However, quiescence is abandoned, and cells return to their normal state, as soon as one of the surrounding nodes becomes free. Proliferation, apoptosis, migration and lysis are modeled as stochastic processes occurring with certain rates. A Poisson process has been assumed for each individual stochastic process and a master equation for the change of the probability of the multi-cellular configuration at time 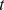 (denoted by the variable 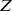) in terms of the multi-cellular configuration in another state 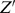 at time 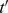 is then used. It reads as follows:
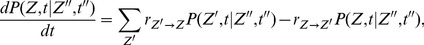
(2)where 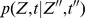 denotes the conditional probability of finding the multi-cellular configuration 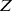 at time 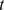 given the configuration was 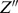 at time 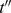, 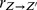 being the transition rate from configuration 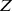 to 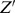. Notice that the master equation (2) is a balance equation. Indeed, the first term on the right of (2) is a gain term that summarizes all transitions that increase the probability of finding the corresponding multi-cellular system in configuration 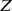. On its turn, the second term in the right describes transitions that move the system away from 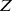, and thus represents a loss term. Equation (2) can be numerically solved if the initial condition 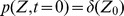 is given, where 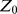 denotes the initial multi-cellular (in our case, tumor cells) configuration. A configuration is determined by the spatial distribution of cells and the state of each cell (proliferating, quiescent, etc.). In our model both CCs and CSCs are able to migrate with the same rate. Migration is mimicked by a hopping process allowing any cell to move from one lattice site to a free neighbor lattice site. In case several free neighbor lattice sites exist, one of them is randomly chosen. In this work we have considered two different migration rates (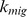), a comparatively low rate (0.025 *h* ^−1^) in the range obtained from the cell diffusion constant [18] as outlined in the Document S1 provided, and a higher rate (1.75 ^h−1^8) as estimated *in vitro* in [43] for a GBM cell line. These will be respectively referred to as low and high migration rates in the sequel. It should be stressed that we only consider here the case where the motion of a cell from one lattice site to another does not depend on the contact energy between neighboring cells, but only on the availability of space. In that case, the higher the migration rate, the stronger the cell dispersion is (see for instance Movies provided as supporting information). If however cell-cell adhesion would be considered, migrating cells would tend to fill holes and cavities [81], and migration will lead instead to tumor compactification. We assume that in our current setting this case is substantially included in very low migration cases.

On the other hand, CCs and CSCs undergo programmed cell death (apoptosis) (see for instance [32]). Disposal of cellular debris resulting from apoptosis is carried out by a lysis process [82], for which a lysis rate 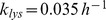 (about 30 *h*) has been assumed. This is about 10-fold less than phagocytosis (digestion of cellular debris by macrophages) observed *in vivo* in [83], but within the range reported for *in vitro* cultures 0.002 *h* ^−1^ for *Hybridoma VO 208* cell line [84] to 0.07 *h* ^−1^ for *Fibrobacter succinogenes* [85].

The master equation (2) has been numerically solved by means of the so-called Gillespie algorithm [86], (also called kinetic Monte-Carlo algorithm or Bortz-Kalos-Lebowitz algorithm [87], see Document S1 for more details). Notice that one advantage of using a lattice model is the possibility of extending the same formalism at larger scales by permitting more than one cell to occupy a single lattice site [80]. In order to simulate the resulting biological effect when a radiation dose *D* is delivered, the surviving fraction is computed for each tumor cell phenotype according to the LQ model (1), taking into account the state of each cell, proliferating or quiescent. Surviving cells are randomly selected out of the total cell number involved. In the next section we will show a typical starting point (about 10^5^ cells) in the computer simulations to be described below, as well as the resulting tumor once a size of about 10^6^ cells has been reached under the kinetic rules just described, and before the radiotherapy treatment is applied. For convenience of the reader, we provide in Table 1 all parameter values used in our mathematical model to simulate the tumor growth and radiation response.

**Table 1:** Model parameters used in computer simulations of tumor growth and radiotherapy treatments.

Description	Symbol	Value/Range	Source
Migration rate	*k_mig_*	0.025 *h* ^−1^/1.75 *h* ^−1^	[18], [43]
Apoptosis rate	*K_apt_*	4.17×10^−4^ *h* ^−1^	[32]
Lysis rate	*K_lys_*	0.035 *h* ^−1^	*(Assumed)*
Radiosensitivity (LQ model)	*α*	0.48 *Gy* ^−1^	[47]
Radiosensitivity (LQ model)	*β*	0.02 *Gy* ^−2^	[47]
Radiosensitivity (LQ model): Proliferating (CC)	*ξ_p_*_1_	1.00	[68], [69]
Radiosensitivity (LQ model): Quiescent (CC)	*ξ_q_*_1_	0.85	[68], [69]
Radiosensitivity (LQ model): Proliferating (CSC)	*ξ_p_*_2_	0.30	[68], [69]
Radiosensitivity (LQ model): Quiescent (CSC)	*ξ_q_*_2_	0.20	[68], [69]
CC cycle duration	*τ_cc_*	26 *h*	[45]
CSC cycle duration	*τ_csc_*	48 *h*, 72 *h*, 96 *h*	*(Assumed)*
Asymmetric division probability (CSC)	*p_a_*	0.75, 0.50, 0.25	*(Assumed)*
Maximum number of cycle divisions (CC)	–	15	[32], [61]

Values for those parameters not found in the literature were assumed (see detailed explanation for lysis rate). In the remaining cases (asymmetric division probability and CSC cycle duration) some values were assumed, and the impact of different parameter sets on the resulting effects was subsequently analyzed.

## Results

**Figure 3:**
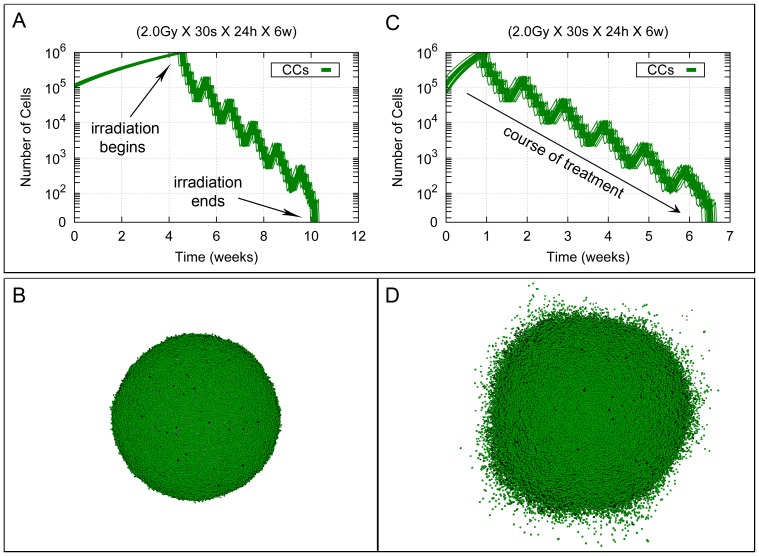
Standard radiotherapy treatment in a homogeneous tumor for the low and high migration cases. Cell growth curves are shown corresponding to homogeneous tumor growth for the low and high migration cases when only CCs are present (see respectively (A) and (C)). Tumor growth is allowed unchecked from a size of about 10^5^ cells until about 10^6^ cells are present, which approximately occurs at day 30 (respectively at day 7) since the beginning of the process. Then, a homogeneous treatment corresponding to 30 sessions of 2.0 *Gy* each is delivered. In all cases, sessions are scheduled along 6 weeks separated by 24 hours intervals except for weekends, where a 72 hours interval is allowed. Radiotherapy treatment is thus completed 40 days afterwards its beginning (about 70 and 47 days since the initial stage respectively). Data corresponding to 20 simulations (with different seeds of a random number generator) are presented. Notice that the vertical coordinate is represented in a logarithmic scale. In (B) and (D) tumor stages are represented when radiation therapy is started (about 10^6^ cells in total) for the low and high migration cases respectively. Depicted in dark and light green are proliferating and quiescent CCs. Dead cells are represented in black. See Movies S1 and S2 for an example of simulations represented in (A) and (C).

As a reference case, we first consider the effect on a fully monoclonal tumor (containing only CCs) of a therapeutic irradiation protocol, consisting of 30 sessions of 2.0 *Gy*, each being homogeneously delivered on the tumor. According to standard radiotherapy scheduling, sessions are distributed into 6 weeks, each week including five sessions from Monday to Friday separated by 24 hours intervals, and with a 72 hours interval from Friday to next Monday in the following week (with weekend interruptions). The total radiation dose delivered with this treatment is thus 60 *Gy*. This is currently considered a standard radiotherapy treatment for most GBM tumors [88], [89], [90]. The corresponding process is illustrated in Figure 3, both for the cases of low and high migration, under our current assumption that migration is not inhibited by cell-cell adhesion. Figure 3 shows that tumor prior to treatment grows from week 1 to approximately week 5 for the low migration case, and just in one week in the case of high migration, until a size of about 10^6^ cells is attained. Notice that the growth time of the tumor decreases with the migration rate due to the decreasing effect of contact inhibition inside the tumor. Then radiation therapy treatment starts, and accordingly tumor cell number diminishes during the first week (with small re-growth between each daily session and weekend interruptions, as represented by the knots in the straight line in the plot, see Figure 3). The pattern just described is reproduced until tumor eradication is achieved at the end of the radiotherapy treatment in these cases.

**Figure 4:**
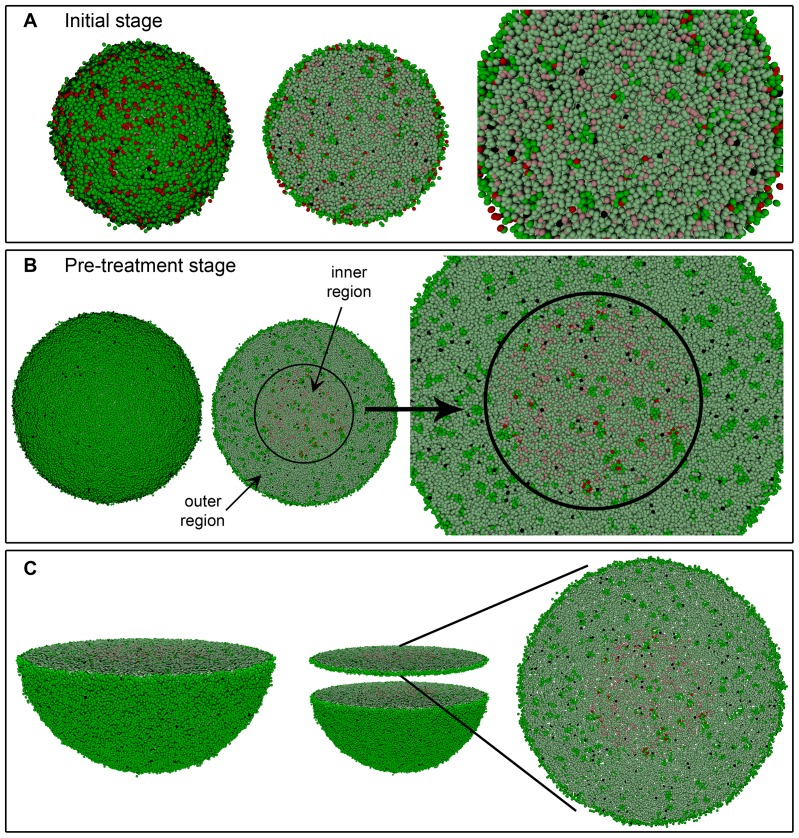
Simulated growth of a heterogeneous tumor with the low migration rate. Depicted in dark and light green (respectively, dark and light red) are proliferating and quiescent CCs (respectively, proliferating and quiescent CSCs). Dead cells are represented in black. (A) An initial stage where about 10^5^ cells, distributed into tumor cell phenotypes CC (85%) and CSC (15%), are present. (B) Tumor stage when radiation therapy is started (about 10^6^ cells in total). In the middle image, the location of the inner region where 100% of CSCs are concentrated is shown for the case when 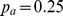 and CSC cycle duration equal to 96 *h*. A 3D transversal cut is performed in the middle of solid figures (A) and (B) (left), so that its interior could be seen (middle and right) respectively. (C) Representation of the transversal cut showed in (B) for a slice of two cell diameters. Notice the little space existing between cells.

**Figure 5:**
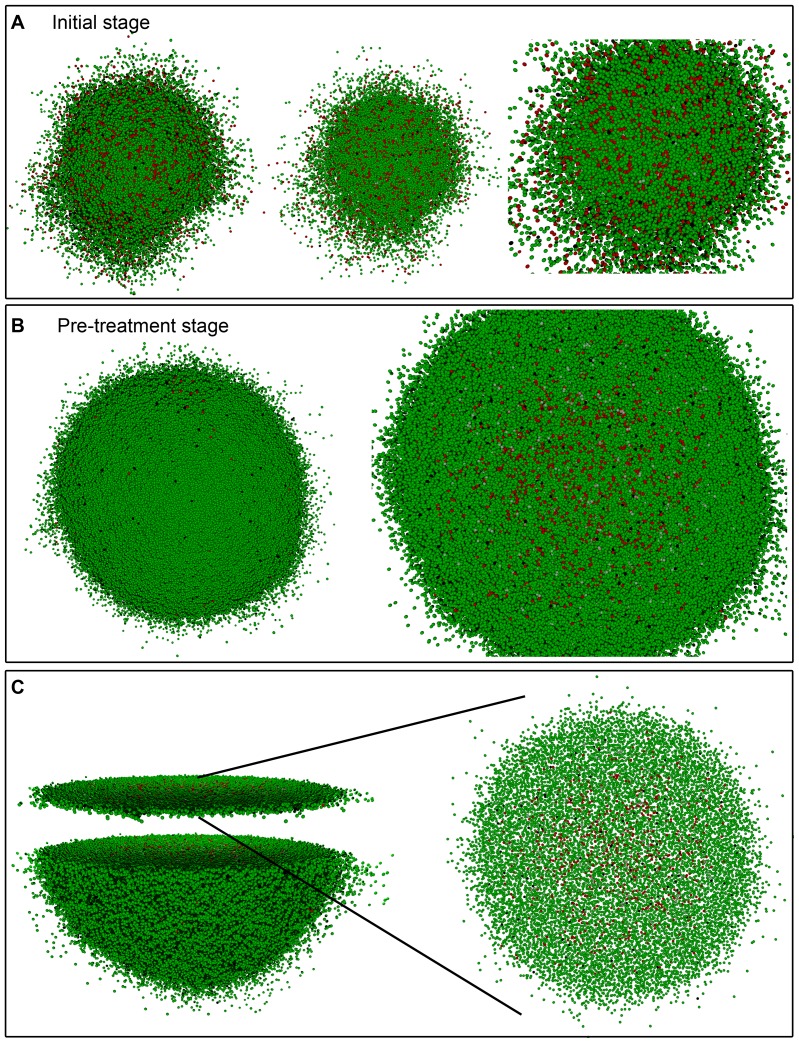
Simulated growth of a heterogeneous tumor with the high migration rate. Depicted in dark and light green (respectively, dark and light red) are proliferating and quiescent CCs (respectively, proliferating and quiescent CSCs). Dead cells are represented in black. (A) An initial stage where about 10^5^ cells, distributed into tumor cell phenotypes CC (85%) and CSC (15%), are present. (B) Tumor stage when radiation therapy is started (about 10^6^ cells in total). In the right image, the spatial distribution of CCs and CSCs is shown for the case when 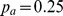 and CSC cycle duration equal to 48 *h*. A 3D transversal cut is performed in the middle of solid figure (B) (left), so that its interior could be seen (right). (C) Representation of the transversal cut showed in (B) for a slice of two cell diameters. Notice the comparatively large (with respect to Figure 4) space observed between cells.

A heterogeneous tumor containing the two tumor cell phenotypes is now considered. Starting from an initial configuration where 10^5^ cells are present, out of which approximately 85% are CCs and 15% are CSCs, tumor growth is allowed until a size of about 10^6^ cells is reached (see Figures 4 and 5). Then the impact of homogeneous and heterogeneous radiation dose distributions is modeled, and computer simulation results are compared in the cases where asymmetric division probabilities *p_a_* for CSCs are 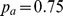, 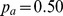 and 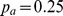, the CSC cycle duration is taken to be 96 *h*, 72 *h* and 48 *h*, and the low and high migration rates are considered. The results obtained will show that the standard irradiation protocol described before fails now to achieve tumor control in any of the cases considered. To compare the dynamics of the tumor resulting after irradiation with respect to its pre-treatment stage, computer simulations are stopped once the pre-treatment population size of about 10^6^ cells is again obtained.

As shown in Figure 4, for 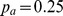, CSC cycle duration equal to 96 *h* and the low migration rate, the more radioresistant tumor cell phenotype CSC is confined within an inner, smaller region when irradiation is started. Such spatial CSCs distribution is neither a priori imposed, nor a consequence of the specific CSC cycle duration or the asymmetric division probability considered (see Table 2). It is due instead to the difference of the CSC and CC cycle durations. Indeed, a robust emerging feature is now observed. Namely, due to asymmetric division CSCs produce a certain fraction of CCs. Both CCs and CSCs then compete for resources including free space at the tumor border [18], [91]. For sufficiently small micro-motility, that competition is controlled by cell replication. As CCs proliferate faster than CSCs, they have a selective advantage in the competition for free space and will eventually outcompete the CSCs in the border region of the tumor, if (as it happens in our case) to achieve such dominance less replications are needed than the maximum number that CCs can perform. The precise timing depends on the relation of the cell cycle duration for CSCs vs. CCs, 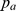, and the fraction of CSCs in the initial population at 10^5^ cells (notice that this fraction would itself be determined by 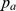 and CSC cycle duration if the 10^5^ cells would already have emerged by replication from a single initial CSC).

**Table 2:** Estimates of the tumor inner region diameter and number of CSCs before irradiation for the low migration case.

	*p_a_* = 0.75		*p_a_* = 0.50		*p_a_* = 0.25	
*τ_csc_*	Diameter	CSCs	Diameter	CSCs	Diameter	CSCs
**96 h**	1426.3 *μm*	16871	1488.6 *μm*	18718	1513.5 *μm*	20448
	[41.07 *μm*]	[56.12]	[43.24 *μm*]	[71.33]	[53.70 *μm*]	[77.30]
**72 h**	1473.6 *μm*	17125	1539.3 *μm*	19366	1587.8 *μm*	21092
	[40.85 *μm*]	[67.50]	[58.67 *μm*]	[102.07]	[40.45 *μm*]	[85.48]
**48 h**	1498.1 *μm*	17785	1595.9 *μm*	19829	1682.2 *μm*	21953
	[47.21 *μm*]	[62.45]	[73.16 *μm*]	[88.98]	[47.74 *μm*]	[140.16]

Diameter is that of an inner sphere where 100% of CSCs are located. CSCs number is computed before radiation therapy treatment starts. Within brackets the corresponding standard deviations are also provided. Data corresponding to 20 simulations (with different seeds of a random number generator) for each case considered. See also Figures in the Document S1 for further details.

Therefore, for a low migration rate, CSCs will be contact-inhibited by the fast proliferating CCs. As a consequence, CSCs will remain confined in an inner region in that case. Actually, on assuming a cell diameter of 20 µ*m*, the diameter of the tumor in all cases is then of about 2680 µ*m* (with a standard deviation of 56 µ*m* over 20 simulations performed for each parameter set considered) and the volume of this inner region where 100% of CSCs are located varies from the 15% to 25% of the total tumor volume, when asymmetric division probability and the CSC cycle duration are allowed to change (see Table 2, and Figures in the Document S1 provided).

**Figure 6:**
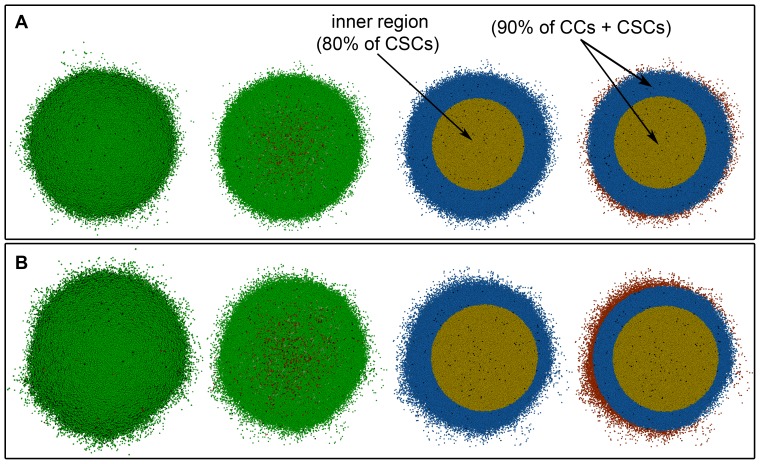
Spatial distribution of CSCs for a heterogeneous tumor with the high migration rate. From left to right tumor stage when radiation therapy is started (about 10^6^ cells in total) with the high migration rate, 3D transversal cut in the middle of the tumor, region where 80% of CSCs are located (yellow) and region where 90% of total cells (CCs and CSCs) are located (yellow and blue). (A) For the case 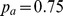 and (B) for 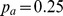 considering CSC cycle duration equal to 48 *h*. Depicted in dark and light green (respectively, dark and light red) are proliferating and quiescent CCs (respectively, proliferating and quiescent CSCs). Dead cells are represented in black.

On the other hand, when the high migration rate is considered, CSCs are not fully concentrated in an inner region of the tumor (see Figure 5 for 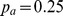 and CSC cycle duration equal to 48 *h*). However, we can define an inner region where at least 80% of CSCs are located (see Figure 6). When asymmetric division probability and CSC cycle duration are allowed to change in the parameter range considered, this inner region approximately represents between 21% to 40% of the volume where 90% of cells, both CCs and CSCs, are located (see Table 3, and Figures in the Document S1 provided). In this case, the diameter of the tumor for all cases is about 5294 µ*m* (with a standard deviation of 778 µ*m* over 20 simulations performed for each parameter set considered). In Tables 2 and 3 the number of CSCs just before treatment starts is shown, so that its dependence with migration rate, asymmetric division probability and CSC cycle duration can be observed. Actually, the number of CSCs existing before treatment starts is a key factor to estimate tumor resistance to radiation therapy, as we will recall below.

**Table 3:** Estimates of the tumor inner region diameter and number of CSCs before irradiation for the high migration case.

	*p_a_* = 0.75		*p_a_* = 0.50		*p_a_* = 0.25	
*τ_csc_*	Diameter	CSCs	Diameter	CSCs	Diameter	CSCs
**96 h**	1857.4 *μm*	20454	1986.9 *μm*	27916	2035.6 *μm*	35087
	[74.72 *μm*]	[256.53]	[51.30 *μm*]	[811.67]	[77.80 *μm*]	[1066.54]
**72 h**	1906.2 *μm*	21178	2043.1 *μm*	28847	2158.7 *μm*	37686
	[54.46 *μm*]	[322.63]	[78.51 *μm*]	[861.47]	[94.21 *μm*]	[859.31]
**48 h**	1983.8 *μm*	21944	2139.3 *μm*	30119	2294.8 *μm*	41629
	[69.64 *μm*]	[506.92]	[81.17 *μm*]	[872.98]	[62.60 *μm*]	[1040.65]

Diameter is that of an inner sphere where 80% of CSCs are located. CSCs number is computed before radiation therapy treatment starts. Within brackets the corresponding standard deviations are also provided. Data corresponding to 20 simulations (with different seeds of a random number generator) for each case considered. See also Figures in the Document S1 for further details.

Bearing these facts in mind, it turns out that tumor control can be obtained in all cases when a radiation boost is applied at such internal regions. More precisely, in the case of low migration we observe that tumor control can be achieved for CSC cycle durations equal to 96 *h* and 72 *h*, when 2.5 *Gy* (for the case 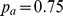), 2.9 *Gy* (for 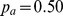) and 3.3 *Gy* (for 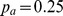) are respectively delivered within the largest inner sphere containing 100% of CSCs, and 2.0 *Gy* is delivered in the rest of the tumor, according to the former standard fractionation protocol (5 days a week along 30 sessions at 24 hours intervals except for weekends). However, when CSC cycle duration is 48 *h*, tumor eradication is not possible with these heterogeneous therapies under the same conditions. In that case, to obtain tumor control, the dose delivered in the inner region has to be raised to 2.7 *Gy*, 3.4 *Gy* and 3.9 *Gy* respectively (see Table 4 and the Document S1 provided). Notice that these radiation doses increase as asymmetric division probability decreases.

**Table 4:** Classification of heterogeneous and homogeneous radiation therapies for the low migration case.

		Heterogeneous therapy		Homogeneous therapy	
*p_a_*	*τ_csc_*	No Control	Control	No Control	Control
0.75	**96 h**	–	2.0 *Gy*–2.5 *Gy* ^(1)^	2.10 *Gy* ^(1)^	2.5 *Gy*
			[63.0 *Gy*]	[63.0 *Gy*]	[75.0 *Gy*]
	**72 h**	–	2.0 *Gy*–2.5 *Gy* ^(2)^	2.10 *Gy* ^(2)^	2.5 *Gy*
			[63.0 *Gy*]	[63.0 *Gy*]	[75.0 *Gy*]
	**48 h**	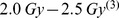	2.0 *Gy*–2.7 *Gy* ^(4)^	2.10 *Gy* ^(3)^/2.12 *Gy* ^(4)^	2.7 *Gy*
		[63.0 *Gy*]	[63.6 *Gy*]	[63.0 *Gy*]/[63.6 *Gy*]	[81.0 *Gy*]
0.50	**96 h**	–	2.0 *Gy*–2.9 *Gy* ^(5)^	2.15 *Gy* ^(5)^	2.9 *Gy*
			[64.5 *Gy*]	[64.5 *Gy*]	[87.0 *Gy*]
	**72 h**	–	2.0 *Gy*–2.9 *Gy* ^(6)^	2.17 *Gy* ^(6)^	2.9 *Gy*
			[65.1 *Gy*]	[65.1 *Gy*]	[87.0 *Gy*]
	**48 h**	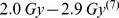	2.0 *Gy*–3.4 *Gy* ^81)^	2.19 *Gy* ^(7)^/2.30 *Gy* ^(8)^	3.4 *Gy*
		[65.7 *Gy*]	[69.0 *Gy*]	[65.7 *Gy*]/[ 69.0 *Gy*]	[102 *Gy*]
0.25	**96 h**	–	2.0 *Gy*–3.3 *Gy* ^(9)^	2.23 *Gy* ^(9)^	3.3 *Gy*
			[66.9 *Gy*]	[66.9 *Gy*]	[99.0 *Gy*]
	**72 h**	–	2.0 *Gy*–3.3 *Gy* ^(10)^	2.27 *Gy*(10)	3.3 *Gy*
			[68.1 *Gy*]	[68.1 *Gy*]	[99.0 *Gy*]
	**48 h**	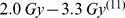	2.0 *Gy*–3.9 *Gy* ^(12)^	2.32 *Gy* ^(11)^/2.47 *Gy* ^(12)^	3.9 *Gy*
		[69.6 *Gy*]	[74.1 *Gy*]	[69.6 *Gy*]/[74.1 *Gy*]	[117 *Gy*]

In all cases, treatment sessions were scheduled along 6 weeks separated by 24 hours intervals except for weekends, where a 72 hours interval is allowed. Data corresponding to 20 simulations (with different seeds of a random number generator) are presented. In the heterogeneous therapies, radiation doses are specified both for the outer (left) and inner (right) tumor regions, each case being indexed from (1) to (12). The averaged dose for any of the previous cases is labeled with the same number in the columns corresponding to homogeneous therapies. Within brackets the total dose of the radiation therapy treatment is also provided. See Tables and Figures in the Document S1 for further details.

**Figure 7:**
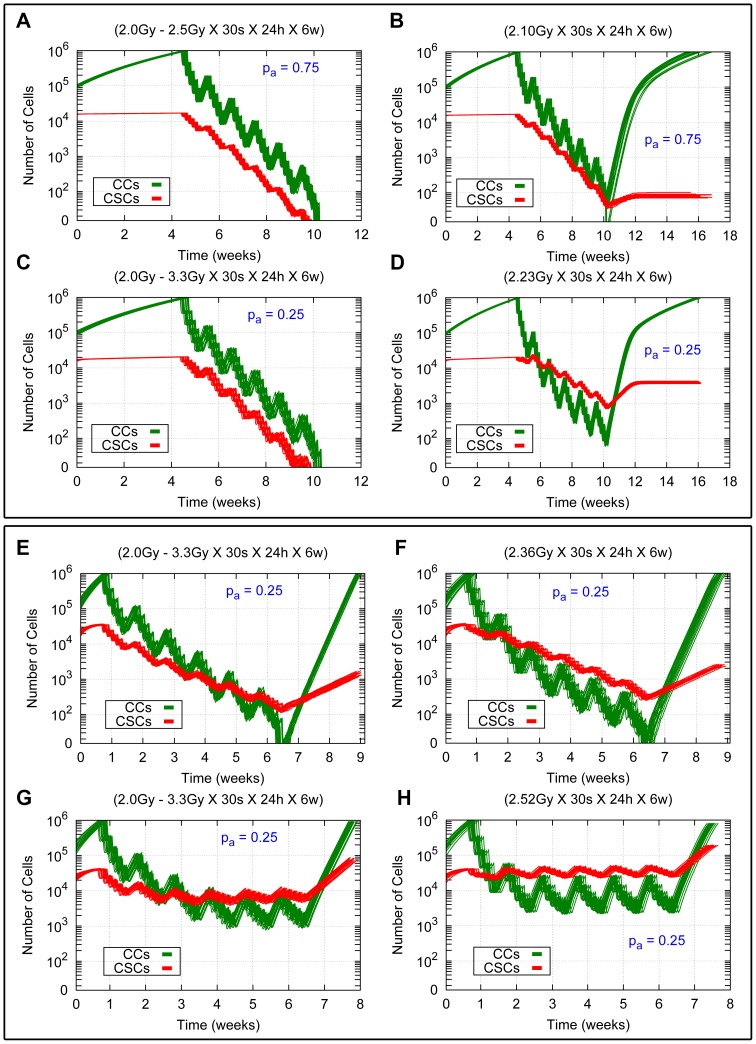
Comparing heterogeneous and averaged homogeneous radiation therapies in a heterogeneous tumor for different model parameters. Cell survival curves for 20 simulations (with different seeds of a random number generator) in the cases 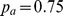 and 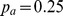 for CSC cycle durations equal to 96 *h* and 48 *h* with the high and low migration rates are shown. The time evolution for CCs and CSCs is represented in green and red respectively. (A, C, E, G) Results for heterogeneous therapies consisting of 2.5 *Gy* and 3.3 *Gy* in the inner sphere and 2.0 *Gy* in the rest of the tumor. (B, D, F, H) Results for the related averaged homogeneous therapies corresponding to 2.10 *Gy*, 2.23 *Gy*, 2.36 *Gy* and 2.52 *Gy* respectively. (A, B, C, D) Results for the cases 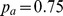 and 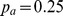 with the low migration rate and CSC cycle duration equal to 96 *h*. (E, F, G, H) Results for the case 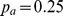 with the high migration rate and CSC cycle durations equal to 93 *h* (E, F) and 48 *h* (G, H). In all cases 30 sessions are scheduled along 6 weeks, separated by 24 hours intervals except for weekends, where a 72 hours interval is allowed. Radiation is applied when the total cell count is about 10^6^ cells. Notice that the vertical coordinate is represented in a logarithmic scale. See Movies S3, S4, S7 and S8 for an example of simulations represented in (B), (D), (G) and (H) respectively.

Let us now consider the same heterogeneous therapies for the case of high migration. We now select an inner region where 80% of CSCs are located. Considering these heterogeneous therapies for each case as before, tumor control is now obtained only for 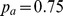 with CSC cycle durations of 96 *h* and 72 h. This is due to the fact that *i)* the high migration rate permits less contact inhibition, which in turn allows for rapid re-growth, and *ii)* there are about 20% of CSCs which only receive a radiation dose of 2.0 *Gy*. Therefore, to obtain tumor control it is not only necessary to increase the radiation dose in the inner region, but also in the outer one (see Table 5 and the Document S1 provided). The radiation doses of the heterogeneous therapies required to obtain tumor control are provided for each case of migration rate considered in Tables 4 and 5. The respective temporal evolution of the number of each tumor cell phenotype is shown in Figure 7 (A), (C), (E) and (G) for different values of asymmetric division probability, migration rate and CSC cycle duration.

**Table 5:** Classification of heterogeneous and homogeneous radiation therapies for the high migration case.

		Heterogeneous therapy		Homogeneous therapy	
*p_a_*	*τ_csc_*	No Control	Control	No Control	Control
0.75	**96 h**	–	2.0 *Gy*–2.5 *Gy* ^(2)^	2.5 *Gy* ^(1)^	2.5 *Gy*
			[63.0 *Gy*]	[63.0 *Gy*]	[75.0 *Gy*]
	**72 h**	–	2.0 *Gy*–2.5 *Gy* ^(2)^	2.11 *Gy* ^(2)^	2.5 *Gy*
			[63.3 *Gy*]	[63.3 *Gy*]	[75.0 *Gy*]
	**48 h**	2.0 *Gy*–2.5 *Gy* ^(3)^	2.2 *Gy*–2.7 *Gy* ^(4)^	2.13 *Gy* ^(3)^/2.33 *Gy* ^(4)^	2.7 *Gy*
		[63.9 *Gy*]	[69.9 *Gy*]	[63.9 *Gy*]/[ 69.9 *Gy*]	[81.0 *Gy*]
0.50	**96 h**	2.0 *Gy*–2.9 *Gy* ^(5)^	2.3 *Gy*–2.9 *Gy* ^(6)^	2.23 *Gy* ^(5)^/ 2.45 *Gy* ^(6)^	2.9 *Gy*
		[66.9 *Gy*]	[73.5 *Gy*]	[66.9 *Gy*]/[73.5 *Gy*]	[87.0 *Gy*]
	**72 h**	2.0 *Gy*–2.9 *Gy* ^(7)^	2.6 *Gy*–2.9 *Gy* ^(8)^	2.25 *Gy* ^(7)^/2.70 *Gy* ^(8)^	2.9 *Gy*
		[67.5 *Gy*]	[81.0 *Gy*]	[67.5 *Gy*]/[81.0 *Gy*]	[87.0 *Gy*]
	**48 h**	2.0 *Gy*–2.9 *Gy* ^(9)^	2.8 *Gy*–3.4 *Gy* ^(10^	2.29 *Gy* ^(9)^/3.00 *Gy* ^(10)^	3.4 *Gy*
		[68.7 *Gy*]	[90.0 *Gy*]	[68.7 *Gy*]/[90.0 *Gy*]	[102 *Gy*]
0.25	**96 h**	2.0 *Gy*–3.3 *Gy* ^(11)^	2.4 *Gy*–3.3 *Gy* ^(12)^	2.36 *Gy* ^(11)^/2.65 *Gy* ^(12)^	3.3 *Gy*
		[70.8 *Gy*]	[79.5 *Gy*]	[70.8 *Gy*]/[79.5 *Gy*]	[99.0 *Gy*]
	**72 h**	2.0 *Gy*–3.3 *Gy* ^(13)^	2.7 *Gy*–3.3 *Gy* ^(14)^	2.43 *Gy*/2.90 *Gy* ^(14)^	3.3 *Gy*
		[72.9 *Gy*]	[87.0 *Gy*]	[72.9 *Gy*]/[87.0 *Gy*]	[99.0 *Gy*]
	**48 h**	2.0 *Gy*–3.3 *Gy* ^(15)^	3.4 *Gy*–3.9 *Gy* ^(16)^	2.52 *Gy* ^(15)^/3.60 *Gy* ^(16)^	3.9 *Gy*
		[75.6 *Gy*]	[108 *Gy*]	[75.6 *Gy*]/[108 *Gy*]	[117 *Gy*]

In all cases, treatment sessions were scheduled along 6 weeks separated by 24 hours intervals except for weekends, where a 72 hours interval is allowed. Data corresponding to 20 simulations (with different seeds of a random number generator) are presented. In the heterogeneous therapies, radiation doses are specified both for the outer (left) and inner (right) tumor regions, each case being indexed from (1) to (16). The averaged dose for any of the previous cases is labeled with the same number in the columns corresponding to homogeneous therapies. Within brackets the total dose of the radiation therapy treatment is also provided. See Tables and Figures in the Document S1 for further details.

It may appear at first glance that the successful results obtained for heterogeneous dosimetries could be a consequence of the overall radiation dose delivered over the tumor being larger than that administered according the standard irradiation protocol (2.0 *Gy* a day, 5 days a week at 24 hours intervals, with weekend interruptions and 60 *Gy* in total). However, heterogeneous dosimetry turns out to be crucial to achieve tumor control. In particular, tumor control fails to be attained when we deliver an averaged homogeneous dose (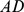), corresponding to the same global radiation energy as in the heterogeneous dosimetry, carried out along a similar scheduling. The corresponding 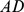 is given by:
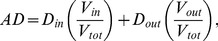
(3)where 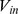, 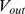 are the volume of the internal sphere and the remaining shell considered; 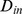, 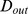 are the radiation doses delivered over the internal and external regions just described, and 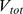 is the total volume of the tumor. In the case of low migration the inner region is that where 100% of CSCs are located (see Table 2 for values of the diameter of this inner region for each case) and the volume of the outer region is computed with respect to the average diameter of the tumor at the beginning of the radiotherapy treatment (2680 µ*m*). However, for the case of high migration the inner region is now selected as that where 80% of CSCs are located (see Table 3 for further details). Indeed, since some cells are now isolated far from the tumor bulk, instead of defining the tumor radius as the distance from its center of mass to the farthest cell, to compute the averaged dose the volume of the tumor is now considered as that of the region where 90% of total cells (CCs and CSCs) are located, where the diameter is about 3120 µ*m* (with a standard deviation of 186 *m* over 20 simulations performed for each parameter set considered). The reason for this assumption is that it will yield a higher 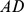 than that obtained when considering the maximum diameter of the tumor (5294 µ*m*), which will extend to regions sparsely occupied by tumor cells. Hence the averaged homogeneous therapies thus derived will deliver higher radiation doses than those that would be obtained if the outer shell were defined as that where all tumor cells are contained.

**Figure 8:**
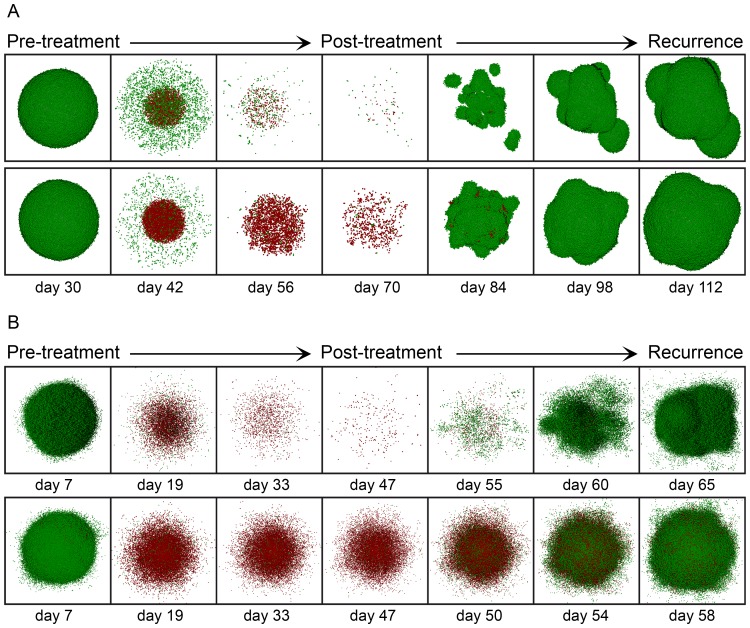
Time evolution of tumor growth during and after averaged homogeneous radiation therapies. (A) A homogeneous dose of 2.10 *Gy* for the case 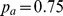 is delivered (Top), and a homogeneous dose of 2.23 *Gy* for 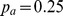 is instead applied (Bottom), assuming in both cases of (A) the low migration rate and CSC cycle duration equal to 96 *h*. (B) A homogeneous dose of 2.36 *Gy* is delivered (Top) and a homogeneous dose of 2.52 *Gy* (Bottom) for the case 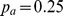 with the high migration rate and CSC cycle durations equal to 96 *h* (Top) and 48 *h* (Bottom). In all cases (A, B) a standard scheduling (30 sessions along 6 weeks separated by 24 hours intervals except for weekends) was applied. From left to right we show in sequential order the tumor before radiotherapy treatment starts, its state after sessions 10, 20 and 30, and three stages corresponding to recurrence during the period covered (where about 10^6^ cells is again obtained). Depicted in dark and light green (respectively, dark and light red) are proliferating and quiescent CCs (respectively, proliferating and quiescent CSCs). Dead cells are not represented.

The averaged dose (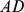) per session according to equation (3) is shown in Tables 4 and 5, for each of heterogeneous therapies described before. These 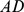 vary from 2.10 *Gy* to 2.32 *Gy* for the low migration case and from 2.10 *Gy* to 2.52 *Gy* in the case of high migration for the asymmetric division probabilities and CSC cycle durations considered. Notice that the total radiation doses delivered by these averaged homogeneous therapies are higher than 60 *Gy* (the value corresponding to the standard irradiation protocol) for all cases. The total radiation doses corresponding to these new dosimetries range between 63.0 *Gy* to 69.6 *Gy* for the case of low migration and 63.0 *Gy* to 75.6 *Gy* for the high migration case. Some of these results are illustrated in Figure 7 (B), (D), (F) and (H). On the other hand, in Figure 8, further details of the time evolution of the tumor colony are provided during and after an homogeneous radiation therapy delivering an 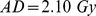 for 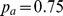 and 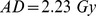 for 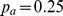 with the low migration rate and CSC cycle duration of 96 *h*. The cases 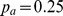 with the high migration rate and CSC cycle durations of 96 *h* and 48 *h*, an 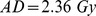 and 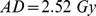 respectively are also included. Notice that in the case of high migration (Figure 8 (B)) more cells remain isolated at the end of the treatment compared with the case of low migration (Figure 8 (A)). In that Figure, when tumor control is not achieved, computer simulations are performed until the surviving tumor reaches a size approximately equal to 10^6^ cells, the number of cells it had before radiotherapy started.

**Figure 9:**
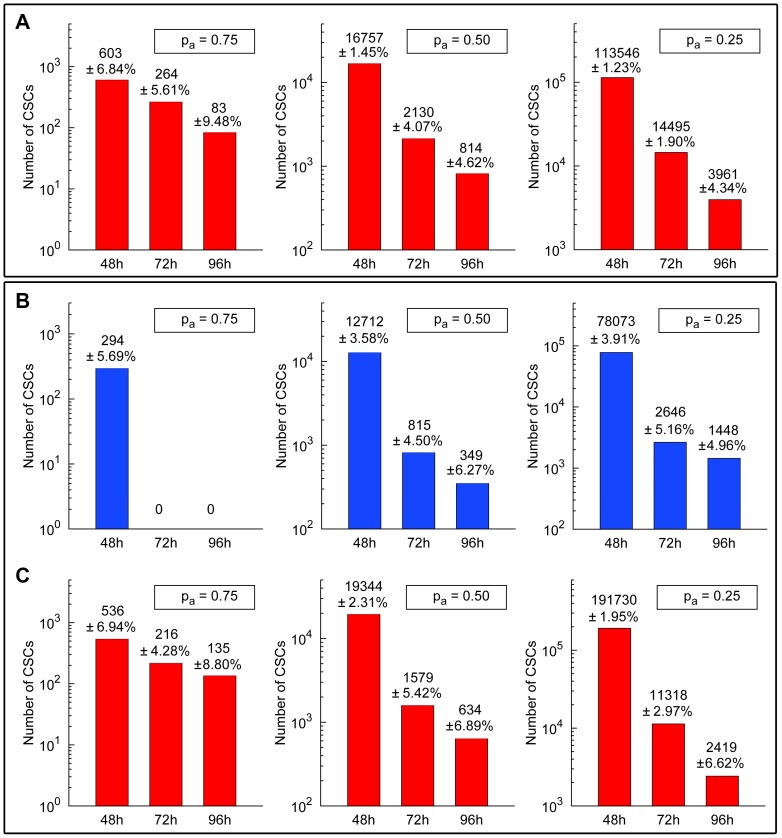
Estimates on the total number of CSCs at the end of the recurrence tumor stage. Number of CSCs at the end of the recurrence tumor stage (where about 10^6^ cells is again obtained) and the corresponding standard deviations after performing 20 simulations in each case (with different seeds of a random number generator) are shown. (A) For averaged homogeneous therapies corresponding to heterogeneous therapies consisting of 2.5 *Gy*, 2.9 *Gy* and 3.3 *Gy* in the inner sphere and 2.0 *Gy* in the rest of the tumor for the cases 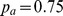, 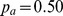 and 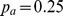 (left, middle, right) assuming the low migration rate and CSC cycle durations equal to 48 *h*, 72 *h* and 96 *h* (see Table 4). (B) For heterogeneous therapies consisting of 2.5 *Gy*, 2.9 *Gy* and 3.3 *Gy* in the inner sphere and 2.0 *Gy* in the rest of the tumor (Top) and the corresponding averaged homogeneous therapies (Bottom) for the cases 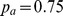, 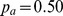 and 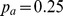 (left, middle, right) with the high migration rate and CSC cycle durations equal to 48 *h*, 72 *h* and 93 *h* (see Table 5). In all cases (A, B, C), a standard scheduling (30 sessions along 6 weeks separated by 24 hours intervals except for weekends) was applied. Notice that the vertical coordinate is represented in a logarithmic scale. See Tables in the Document S1 for further details and Movies S3, S4, S5, S6, S7 and S8 for some examples of simulations represented in (A), (B) and (C).

Tables 4 and 5 reveal that tumor recurrence occurs in all cases for a homogeneous therapy delivering the corresponding average dose (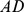). Besides, the number of CSCs in the tumor at the end of the radiotherapy treatment decreases with 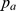 and CSC cycle duration (see Figure 9, and Tables in the Document S1 provided). In the case of low migration, for the heterogeneous therapies failing to achieve tumor control, the number of CSCs remaining alive at the end of the recurrence tumor stage is 107, 1785 and 4457 respectively, with the corresponding standard deviations being 8.53, 78.31 and 232.67 (see Figure 9 (A) to compare with the corresponding averaged homogeneous therapies). These values correspond to the cases 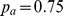, 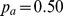 and 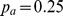 with a CSC cycle duration of 48 *h*. In Figure 9 (B), the number of CSCs at the end of the recurrence tumor stage is provided in the case of high migration for the heterogeneous therapies delivering 2.5 *Gy* (for the case 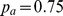), 2.9 *Gy* (for 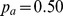) and 3.3 *Gy* (for 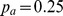) in the inner region, and 2.0 *Gy* in the rest of the tumor. Notice that, even when tumor control cannot be achieved with the heterogeneous therapies, the corresponding averaged homogeneous therapies always have more CSCs at the end of the recurrence tumor stage (see Figure 9 (C)). Moreover, in some cases that number of CSCs is larger than before the treatment started, resulting in more radioresistant tumors after treatment (see Document S1 for further details).

Thus, to achieve full eradication of a tumor consisting of two different tumor cell phenotypes, heterogeneous dosimetry is crucial. Actually, the choice of a minimal radiation dose sufficient to achieve tumor control depends on the value of 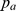, the CC and CSC cycle durations and on the internal spatial distribution of CSCs. In Tables 4 and 5, we describe the heterogeneous radiation therapies needed to achieve tumor control, and the corresponding averaged homogeneous therapies are also provided. Interestingly, the corresponding averaged homogeneous therapies in each case fail to obtain tumor control (see also Tables in the Document S1 provided). Moreover, homogeneous therapies needed to obtain tumor control are also provided in Tables 4 and 5. One readily sees that in all cases higher total radiation doses are needed for homogeneous than for heterogeneous therapies.

**Figure 10:**
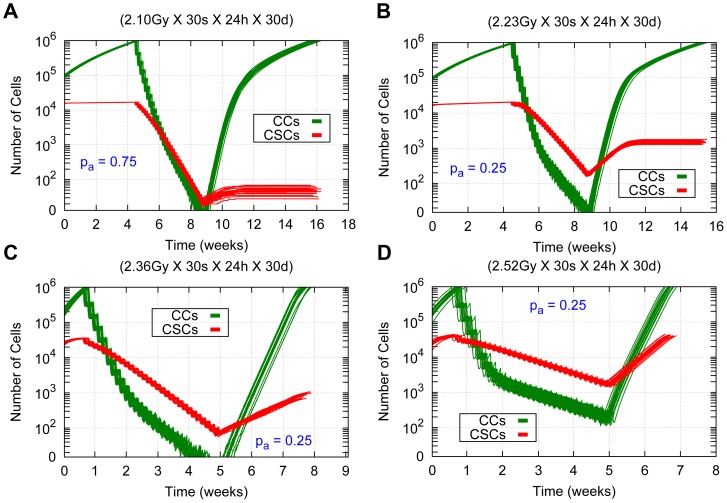
Comparing averaged homogeneous radiation therapies without weekend interruptions. Cell survival curves for 20 simulations (with different seeds of a random number generator) are shown. (A) Averaged dosimetries consisting of 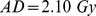 for 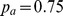 and (B) 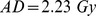 for 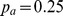, both for the low migration case and CSC cycle duration equal to 96 *h*. (C, D) Averaged homogeneous therapies consisting of 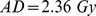 and 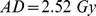 for the case 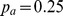 with the high migration rate and CSC cycle durations equal to 96 *h* and 48 *h* respectively. The time evolution of CCs and CSCs are represented in green and red respectively. In all cases (A, B, C, D), sessions were scheduled 7 days a week separated by 24 hours intervals along 30 sessions (without weekend interruptions). Notice that the vertical coordinate is represented in a logarithmic scale. See Movie S9 for an example of simulations represented in (D).

On the other hand, considering that for all choices of model parameters the 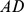 is higher than 2.0 *Gy*, this implies that tumor recurrence will also occur for the standard irradiation protocol (2.0 *Gy* a day, 5 days a week at 24 hours intervals, with weekend interruptions and 6. *Gy* in total) for each case of 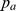, migration rate and CSC cycle duration considered. In terms of the number of remaining CSCs after treatment is completed, recurrence is certainly weaker when 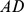 is delivered than for the standard fractionation protocol, as one could expect from the comparative increase in radiation delivery. Moreover, tumor control cannot be achieved for each case of 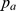, migration rate and CSC cycle duration considered even when the homogeneous therapy delivering the average radiation dose is rescheduled in 7 days a week along 30 sessions at 24 hours intervals, without weekend interruptions (see Figure 10 for some examples of averaged homogeneous therapies with this fractionation protocol).

Since in many clinical scenarios radiation doses are mostly limited by damage inflicted at neighboring organs at risk and healthy tissues (see [92], [93] and [94]), it is important to estimate what amount of tumor control can be achieved when radiation dose distributions are kept as low as possible. In what follows, we shall consider a heterogeneous therapy for which the average radiation dose is approximately equal to 60 *Gy*, and a case of hyperfractionation (a type of scheduling consisting of comparatively many sessions, usually more than 1 per day, with low radiation doses [95]); cf. [96] for a specific study on GBM tumors. We shall see that in these cases a heterogeneous radiation dose distribution also yields better results than its averaged homogeneous equivalent, even when tumor control is not achieved.

**Figure 11:**
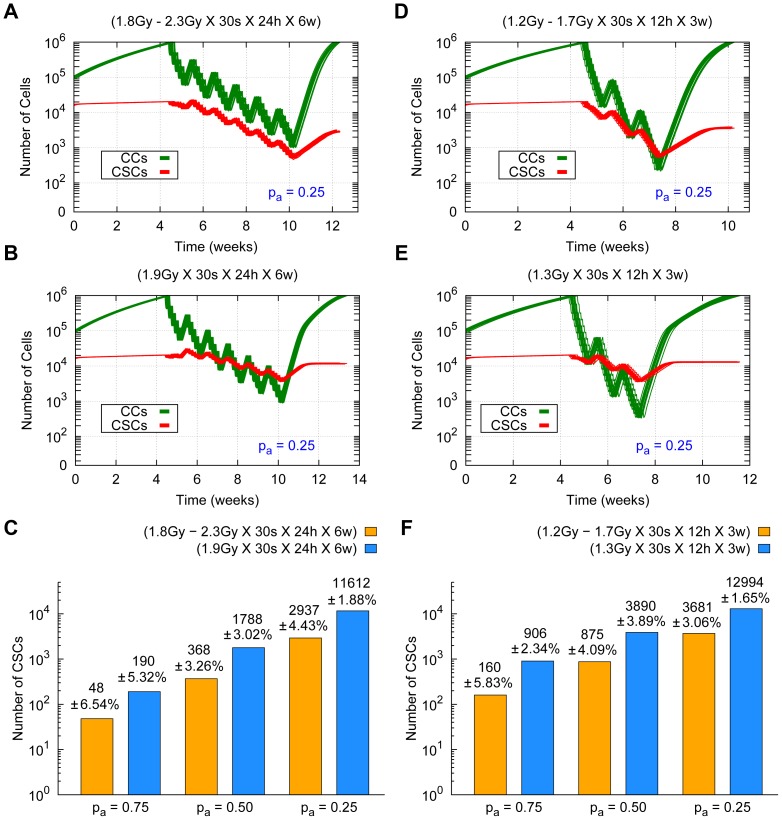
Comparing the effects of lower radiation dosimetries with and without hyperfractionation. Cell survival curves for 20 simulations (with different seeds of a random number generator) are shown in the case 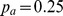 for the low migration case and CSC cycle duration equal to 96 *h*. (Top) From left to right heterogeneous therapies consisting of 2.3 *Gy* (A) and 1.7 *Gy* (D) in the inner sphere, and 1.8 *Gy* (A) and 1.2 *Gy* (D) in the rest of the tumor respectively. (Middle) From left to right the averaged homogeneous therapies corresponding to 1.9 *Gy* (B) and 1.3 *Gy* (E) are represented. Radiation dose delivery been made according to 5 days a week, 30 sessions in total, at 24 hours (A, B) and at 12 hours (D, E) intervals with weekend interruptions. The time evolution of CCs and CSCs is represented in green and red respectively. (Bottom) Number of CSCs and the corresponding standard deviations at the end of the recurrence tumor stage (where about 10^6^ cells is again obtained) for heterogeneous (yellow) and averaged homogeneous (blue) radiation therapies (C, F). Notice that the vertical coordinate is represented in a logarithmic scale. See Movie S10 for an example of simulations represented in (E).

Consider first the case of low migration, CSC cycle duration equal to 96 *h* and where the value of the total radiation dose is a bit less than 60 *Gy* for heterogeneous therapies consisting of 2.3 *Gy* for 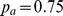, 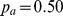 and 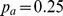 within the largest inner sphere containing 100% of CSCs and 1.8 *Gy* in the rest of the tumor delivered 5 days a week along 30 sessions at 24 hours intervals with weekend interruptions. Computer simulations show that these radiation dosimetries fare better than their averaged homogeneous versions, where total 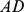 lies between 56 *Gy* and 58 *Gy* for the values of 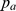 considered (see Figure 11 (A), (B) and (C)). On the other hand, similar results can be obtained for a lower total radiation dose when the time lapse between sessions is also shortened. More precisely, consider the same cases but now for heterogeneous therapies consisting of 1.7 *Gy* within the largest inner sphere containing 100% of CSCs and 1.2 *Gy* in the rest of the tumor, delivered in two sessions per day, 5 days a week along 30 sessions 12 hours intervals, with weekend interruptions. While tumor control is not achieved, tumor radioresistance, measured in terms of the final proportion of CSCs, turns out to be lower for heterogeneous dosimetries than their averaged versions (see Figure 11 (D), (E) and (F)). Notice that in this case, the total averaged doses delivered by the heterogeneous dosimetries considered are much smaller than 60 *Gy* (between 39 *Gy* and 39 *Gy* for the values of 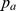 considered).

Figure 11 shows that there is tumor recurrence in all cases. However, it turns out that the number of CSCs remaining at the end of the recurrence tumor stage after radiation therapy is lower than that existing prior to therapy in all cases. Thus tumors surviving this therapy can be considered as less radioresistant than they were before radiation therapy started. An inspection of Figure 11 (C) and (F) quickly shows that in our current cases heterogeneous therapies yield better results than its averaged homogeneous counterparts previously discussed. For completeness, we provide estimates on the total number of CSCs after treatments are concluded and recurrence appears for each case of 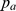 considered (see Figure 11).

We conclude this Section by remarking on the dependence of our model of tumor growth, and the results derived from its analysis, on data and parameters assumed.

To begin with, our results are not restricted to the figure selected (15%) for the proportion of CSCs within the tumor at the initial stage. In fact, they continue to hold as long as the more radioresistant tumor cell phenotype CSC represents a small percentage of the total tumor cell count. A particularly interesting limit case is that when tumor growth starts from a single CSC. Then for each value of 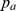 (0.75, 0.50, 0.25), CSC cycle duration (96 *h*, 72 *h* and 48 *h*) and migration rate 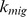 (0.025 *h* ^−1^, 1.75 *h* ^−1^) considered, the number of CSCs present when tumor has reached a size of about 10^6^ cells (just before radiation treatment starts) is much smaller than that corresponding to the cases considered in this work. For instance, in the case of CSC cycle duration equal to 48 *h*, 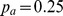, for the low and high migration cases the number of CSCs before the treatment starts is about 5956 and 14316 (with standard deviations of 129 and 530 over 20 simulations performed for each parameter set considered) respectively. The corresponding values for the case considered in this work are 21953 and 41629 respectively (see Tables 2 and 3). Moreover, the internal region where CSCs remain confined is smaller (1120 µ*m* and 1840 µ*m* with standard deviations of 39.7 µ*m* and 71.4 µ*m* over 20 simulations performed for each parameter set considered, respectively) than that reported in Tables 2 and 3. Hence any radiotherapy treatment that achieves tumor control in our case also does so for tumors staring from a single CSC under assumptions above.

On the other hand, we have made use of the assumption that the duration of cell cycle for CSCs is significantly longer than that of CCs, a hypothesis commonly assumed in the literature (cf. [16], [48], [49], [50], [51], [52], [53], [54], [55] and [56]). This fact notwithstanding, our model can be used to examine also the opposite situation, that is the case where CSC cycle duration is equal or smaller than that of ordinary CCs. As an example, we have considered the cases where CSC cycle lasts 26 hours (respectively 18 hours), which is equal to (respectively less than) the 26 hours cell cycle selected for CCs. As one can expect, the inner core where most CSCs remain concentrated is now larger than when slow-cycling CSCs is assumed. In particular, in the case of low migration and for a CSC cycle duration of 26 *h*, such internal regions (where 100% of CSCs are located) range from 20% to 83% of the total tumor volume for values of 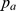 equal to 0.75, 0.50 and 0.25. Besides, when CSC cycle duration is taken to be 18 *h* that internal volume further expands, ranging now between 23% and 100% of the total tumor volume. Additional details, including the number of CSCs present when tumor size reaches about 10^6^ cells and the case of high migration, are provided (see Document S1).

A case which has not been addressed in this work is cancer cell plasticity, a hypothesis that has been advanced to better understand the onset of resistance after therapy; see for instance [97], [98] and [99]. According to this scenario, in addition to CSCs giving raise to CCs by asymmetric division, a (supposedly small) percentage of CCs may transform to a CSC phenotype, possibly as a reaction to radiation therapy. Although little quantitative information about this process seems to be available as yet, including such type of process in our model is possible. To support this statement, we have studied a particular example. Specifically, we have examined a model situation where a small percentage of CCs are transformed to CSCs along the radiation treatment. As expected, any increase in the number of CSCs results in increased malignancy, measured in terms of higher resistance to radiation therapy. However, our conclusion that heterogeneous, tumor-adapted radiation therapies fare better than their corresponding averaged homogeneous versions continues to hold. Details on this study can be found in the Document S1 provided.

## Discussion

Tumor heterogeneity is being increasingly recognized as a key obstacle to achieve successful tumor control, either by means of radiotherapy, chemotherapy or through the use of combined therapies. Indeed, it is well known that tumors at an advanced stage contain different tumor subpopulations, which might have been generated as a consequence of sequential mutations of one initial clonogenic line, or could result from the presence of Cancer Stem Cells. Moreover, it is expected that such cell phenotypes may considerably differ in their biological and radiobiological properties, and in particular in their resistance to radiation (cf. for instance [15], [6], [60], [8], [100], [101]).

Accordingly, it has been proposed that the clinical prognosis of a given tumor would critically depend on the information that may be gathered about its internal heterogeneity, and more precisely, about the identification of regions within it with different sensitivities to a given therapy. In principle, once the spatial distribution of the various cell phenotypes coexisting in a tumor is known, and the resistance to therapy of each of these regions had been estimated, personalized strategies complementary to (or as a substitute to) surgery, could be designed to improve chances of clinical success. The latter can be understood either as total tumor eradication (the standard paradigm as of today) or as achieving instead a stable, chronically-controlled tumor burden where less aggressive lines keep at bay more resistant ones [102]. In either case, significant information towards a personalized treatment would be inferred from knowledge of the internal, non-homogeneous structure of a tumor and the resulting differences in therapy resistance corresponding to the regions thus identified.

Unfortunately, to this day only partial information can be derived about tumor heterogeneity by means of non-invasive medical imaging modalities. Currently available information can be mainly used to distinguish various level of oxygen pressure within the tumor related to hypoxia processes [9], necrotic areas or highly proliferating regions detected by means of PET techniques [103]. While undoubtedly important, such information is not enough to design personalized therapies whose results could significantly improve those obtained by current procedures.

In this work, we have proposed a mathematical model of tumor growth to gain insight about two key issues: how heterogeneity unfolds in a growing tumor, and what type of radiation dosimetry is best suited to achieve control in heterogeneous tumors. Concerning the first issue, we noticed that substantial information about the evolution of spatial heterogeneity within a tumor can be retrieved from knowledge of a few key biological properties of the tumor cell phenotypes involved. In particular, we have shown in the first place that a difference in cell cycle duration between a majority of ordinary cancer cells (CCs) and a minority of comparatively slow-cycling cancer stem cells (CSCs) leads to a concentration of CSCs in regions that can be a priori estimated. In the cases just discussed, such regions consist in an internal core within an expanding tumor, but the result would apply to other geometries as well. In particular, it can be extended to larger tumors with corrugated shapes and boundaries.

We have already mentioned that our key assumption that CSCs have longer replication times than CCs is commonplace in the literature (see for instance [16], [48], [49], [50], [51], [52], [53], [54], [55] and [56]). As a matter of fact, such assumption is naturally associated to the consideration of CSCs as a subpopulation of tumor cells which is able to rescue tumor growth after therapies have been delivered. This is related to the fact that standard radiation therapies preferentially target dividing cells (which are more radiosensitive), and thus spare those that have slower cycles or remain quiescent. Notice that the cell cycle duration could in principle be estimated, at least *in vitro*, for all cell phenotypes known to appear in a given tumor. Importantly, the spatial heterogeneity pattern thus observed does not depend so much on the precise values of such biological parameters, but rather on the fact that they are significantly different for the tumor cell phenotypes involved. As a consequence, the result obtained is robust with respect to fluctuations in cell cycle duration due to systemic factors.

A second result obtained is that, once information about functional heterogeneity had been obtained, tumor-tailored radiation dosimetries can be designed to improve the treatment outcome. We have shown that heterogeneous radiation dosimetries do better than homogeneous ones when regions occupied by different radioresistant tumor subpopulations can be identified, and this is in particular the case when more radioresistant phenotypes are assumed to replicate at a lower pace than less resistant ones. Interestingly, this result holds when the more radioresistant phenotype is allowed to sustain unlimited replication as in the case of CSCs, as opposed to the limited number of replications commonly assumed on CCs. The previous statement holds true, no matter the type of scheduling considered (with or without weekend interruptions) or the precise result pursued, being it total tumor eradication, controlled recurrence or palliative treatment. We believe that the comparative advantage of heterogeneous radiation dose distributions deserves some consideration, since to this day homogeneous dosimetries continue to be those being commonly implemented worldwide.

It is worth to stress that our model is quite robust with respect to changes in data and parameter values. In particular, our conclusions remain in force when CSC and CC cycle durations undergo considerable changes, as long as CSC cycle is significantly slower than that of CCs. Also, CCs and CSCs migration rates are allowed to undergo substantial changes (corresponding for instance to slow and fast migration processes) as far as both tumor cell phenotypes share a similar migration rate. Moreover, our results continue to hold when changes in the choice of the radiosensitivity parameters 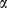 and 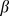 in (1) are allowed, or when different fractions of the minority phenotype (CSC) are assumed. For instance, our results are not confined to the choice made for the assumed percentage (15%) of CSCs present at an early stage of tumor growth. They continue to hold if a different figure for that proportion is taken, as long as CSCs remain a small fraction of the total tumor population at that stage.

On the other hand, cancer cell plasticity has recently received considerable attention ([97], [98] and [99]). We have studied a particular example where in addition to CCs being generated by CSCs with asymmetric division, a small percentage of CCs are transformed to CSCs as a consequence of radioresistance to therapy. Our conclusion that heterogeneous, tumor-adapted radiation therapies fare better than their corresponding averaged homogeneous versions continues to hold in this case.

We conclude by discussing on some of the limitations of this work, as well as on possible extensions thereof. To begin with, we are aware that more research is needed to understand the possible mechanisms that can be responsible for slow cycling of CSCs. Particularly relevant in this context would be to ascertain if slow cycling can, at least in some cases, be established as an intrinsic property of CSCs or if it could alternatively be induced by systemic feedback in the course of tumor growth. Interestingly, even if CSCs are assumed to cycle faster than CCs, our model still shows that heterogeneous dosimetries adapted to the resulting tumor heterogeneity continue to outperform standard homogenous therapies currently in use.

A general conclusion that follows from our study is that detailed information about intratumoral heterogeneity is needed in order to implement efficient dose-painting techniques in clinical practice. In particular, in this work a clear dependence on tumor heterogeneity of the radiation doses needed to achieve tumor control has been obtained. In fact, the inner tumor regions where more radioresistant tumor cell phenotype remains confined are shown to strongly depend on CSC cycle duration and their probability of asymmetric division. In the particularly unfavorable assumption of fast CSCs cycling, this region may rank from 20% to 100% of the total tumor volume. In this latter situation, a worst-case scenario corresponding to a high and homogeneous radiation dose being prescribed and only limited by neighboring organs at risk tolerance, is recovered that corresponds to current clinical practice. Our results suggest that such situation could be considerably improved in many cases if and when sufficient information about key different biological and radiobiological properties of the tumor cell phenotypes present in a given tumor is available, be it either by estimating patient-specific parameters or by means of medical imaging techniques.

Finally, it looks feasible from a mathematical viewpoint to address within this framework situations where larger tumors are considered, a number of cell phenotypes coexist there due to mutations, and other effects (immune response, nutrient limitation, etc.) are accounted for. For example, the modeling framework selected in this work permits simulations to be scaled up to cubic centimeter sizes, though at the expense of lower spatial and functional resolution, and more computing resources. It can also be used to construct hybrid models, zooming in at the cell scale in regions of interest. In particular, we have chosen to represent each cell individually to exclude averaging effects when studying the relation between tumor heterogeneity and simulated radiation outcomes. We hope that this work could provide a starting point towards the study of the more general situations described above.

## Supporting Information

Document S1 **Details of computer simulations of the model of tumor growth and additional results.**
(PDF)

Movie S1 **Time evolution of a homogeneous tumor growth where only CCs are present for the low migration case.** Tumor growth is allowed unchecked from a size of about 10^5^ cells until about 10^6^ cells are present. Then, a standard homogeneous radiation therapy corresponding to 30 sessions of 20 *Gy* each is delivered. Sessions from Monday to Friday are scheduled along 6 weeks, separated by 24 hours intervals except for weekends, where a 72 hours interval is allowed. Depicted in dark and light green (respectively, dark and light red) are proliferating and quiescent CCs (respectively, proliferating and quiescent CSCs). Dead cells are represented in black. See Figure 3 (A, B) in the article.
(MP4)

Movie S2 **Time evolution of a homogeneous tumor growth where only CCs are present for the high migration case.** Tumor growth is allowed unchecked from a size of about 10^5^ cells until about 10^6^ cells are present. Then, a standard homogeneous radiation therapy corresponding to 30 sessions of 2.0 *Gy* each is delivered. Sessions from Monday to Friday are scheduled along 6 weeks, separated by 24 hours intervals except for weekends, where a 72 hours interval is allowed. Depicted in dark and light green (respectively, dark and light red) are proliferating and quiescent CCs (respectively, proliferating and quiescent CSCs). Dead cells are represented in black. See Figure 3 (C, D) in the article.
(MP4)

Movie S3
**Time evolution of a heterogeneous tumor growth (where CCs and CSCs are present) for the low migration case with** 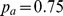 **and CSC cycle duration equal to 96** ***h*** **.** Tumor growth is allowed unchecked from a size of about 10^5^ cells until about 10^6^ cells are present. Then, a homogeneous radiation therapy consisting of 2.10 *Gy* in the tumor is delivered. Treatment sessions are scheduled along 6 weeks, separated by 24 hours intervals except for weekends, where a 72 hours interval is allowed. Depicted in dark and light green (respectively, dark and light red) are proliferating and quiescent CCs (respectively, proliferating and quiescent CSCs). Dead cells are represented in black. See Figure 7 (B) in the article.(MP4)

Movie S4
**Time evolution of a heterogeneous tumor growth (where CCs and CSCs are present) for the low migration case with** 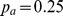 **and CSC cycle duration equal to 63** ***h*** **.** Tumor growth is allowed unchecked from a size of about 10^5^ cells until about 10^6^ cells are present. Then, a homogeneous radiation therapy consisting of 2.23 *Gy* in the tumor is delivered. Treatment sessions are scheduled along 6 weeks, separated by 24 hours intervals except for weekends, where a 72 hours interval is allowed. Depicted in dark and light green (respectively, dark and light red) are proliferating and quiescent CCs (respectively, proliferating and quiescent CSCs). Dead cells are represented in black. See Figure 7 (D) in the article.(MP4)

Movie S5
**Time evolution of a heterogeneous tumor growth (where CCs and CSCs are present) for the low migration case with** 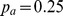 **and CSC cycle duration equal to 48** ***h*** **.** Tumor growth is allowed unchecked from a size of about 10^5^ cells until about 10^6^ cells are present. Then, a heterogeneous radiation therapy consisting of 3.3 *Gy* in the inner sphere and 2.0 *Gy* in the rest of the tumor is delivered. Treatment sessions are scheduled along 6 weeks, separated by 24 hours intervals except for weekends, where a 72 hours interval is allowed. Depicted in dark and light green (respectively, dark and light red) are proliferating and quiescent CCs (respectively, proliferating and quiescent CSCs). Dead cells are represented in black. See Tables 2 and 4 in the article for further details.(MP4)

Movie S6
**Time evolution of a heterogeneous tumor growth (where CCs and CSCs are present) for the low migration case with** 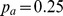 **and CSC cycle duration equal to 48** ***h*** **.** Tumor growth is allowed unchecked from a size of about 10^5^ cells until about 10^6^ cells are present. Then, a homogeneous radiation therapy consisting of 2.32 *Gy* in the tumor is delivered. Treatment sessions are scheduled along 6 weeks, separated by 24 hours intervals except for weekends, where a 72 hours interval is allowed. Depicted in dark and light green (respectively, dark and light red) are proliferating and quiescent CCs (respectively, proliferating and quiescent CSCs). Dead cells are represented in black. See Tables 2 and 4 in the article for further details.(MP4)

Movie S7
**Time evolution of a heterogeneous tumor growth (where CCs and CSCs are present) for the high migration case with** 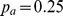 **and CSC cycle duration equal to 48** ***h*** **.** Tumor growth is allowed unchecked from a size of about 10^5^ cells until about 10^6^ cells are present. Then, a heterogeneous radiation therapy consisting of 3.3 *Gy* in the inner sphere and 2.0 *Gy* in the rest of the tumor is delivered. Treatment sessions are scheduled along 6 weeks, separated by 24 hours intervals except for weekends, where a 72 hours interval is allowed. Depicted in dark and light green (respectively, dark and light red) are proliferating and quiescent CCs (respectively, proliferating and quiescent CSCs). Dead cells are represented in black. See Figure 7 (G) in the article.(MP4)

Movie S8
**Time evolution of a heterogeneous tumor growth (where CCs and CSCs are present) for the high migration case with** 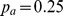 **and CSC cycle duration equal to 48** ***h*** **.** Tumor growth is allowed unchecked from a size of about 10^5^ cells until about 10^6^ cells are present. Then, a homogeneous radiation therapy consisting of 2.52 *Gy* in the tumor is delivered. Treatment sessions are scheduled along 6 weeks, separated by 24 hours intervals except for weekends, where a 72 hours interval is allowed. Depicted in dark and light green (respectively, dark and light red) are proliferating and quiescent CCs (respectively, proliferating and quiescent CSCs). Dead cells are represented in black. See Figure 7 (H) in the article.(MP4)

Movie S9
**Time evolution of a heterogeneous tumor growth (where CCs and CSCs are present) for the high migration case with** 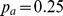 **and CSC cycle duration equal to 48** ***h*** **.** Tumor growth is allowed unchecked from a size of about 10^5^ cells until about 10^6^ cells are present. Then, a homogeneous radiation therapy consisting of 2.52 *Gy* in the tumor is delivered. Treatment sessions are scheduled 7 days a week separated by 24 hours intervals along 30 sessions (without weekend interruptions). Depicted in dark and light green (respectively, dark and light red) are proliferating and quiescent CCs (respectively, proliferating and quiescent CSCs). Dead cells are represented in black. See Figure 10 (D) in the article.(MP4)

Movie S10
**Time evolution of a heterogeneous tumor growth (where CCs and CSCs are present) for the low migration case with** 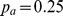 **and CSC cycle duration equal to 96** ***h*** **.** Tumor growth is allowed unchecked from a size of about 10^5^ cells until about 10^6^ cells are present. Then, a homogeneous radiation therapy consisting of 1.30 *Gy* in the tumor is delivered. Treatment sessions are scheduled along 3 weeks, 5 days a week separated by 12 hours intervals except for weekends, where a 72 hours interval is allowed. Depicted in dark and light green (respectively, dark and light red) are proliferating and quiescent CCs (respectively, proliferating and quiescent CSCs). Dead cells are represented in black. See Figure 11 (E) in the article.(MP4)

## References

[1] Moding EJ, Kastan MB, Kirsch DG (2013) Strategies for optimizing the response of cancer and normal tissues to radiation. Nat Rev Drug Discov 12: 526–42. 23812271 10.1038/nrd4003 PMC3906736

[2] Amini A, Lou F, Correa AM, Baldassarre R, Rimner A, et al (2013) Predictors for locoregional recurrence for clinical stage iii-n2 non-small cell lung cancer with nodal downstaging after induction chemotherapy and surgery. Ann Surg Oncol 20: 1934–40. 23263700 10.1245/s10434-012-2800-x PMC3656229

[3] Paulsson AK, McMullen KP, Peiffer AM, Hinson WH, Kearns WT, et al.. (2012) Limited margins using modern radiotherapy techniques does not increase marginal failure rate of glioblastoma. Am J Clin Oncol 1. 10.1097/COC.0b013e318271ae03 PMC4485493 23211224

[4] Gerlinger M, Rowan AJ, Horswell S, Larkin J, Endesfelder D, et al (2012) Intratumor heterogeneity and branched evolution revealed by multiregion sequencing. N Engl J Med 366: 883–92. 22397650 10.1056/NEJMoa1113205 PMC4878653

[5] Sottoriva A, Spiteri I, Piccirillo SG, Touloumis A, Collins VP, et al (2013) Intratumor heterogeneity in human glioblastoma reects cancer evolutionary dynamics. Proc Natl Acad Sci U S A 110: 4009–14. 23412337 10.1073/pnas.1219747110 PMC3593922

[6] Marusyk A, Polyak K (2010) Tumor heterogeneity: causes and consequences. Biochim Biophys Acta 1805: 105–17. 19931353 10.1016/j.bbcan.2009.11.002 PMC2814927

[7] Marusyk A, Almendro V, Polyak K (2012) Intra-tumour heterogeneity: a looking glass for cancer? Nat Rev Cancer 12: 323–34. 22513401 10.1038/nrc3261

[8] Bao S, Wu Q, McLendon RE, Hao Y, Shi Q, et al (2006) Glioma stem cells promote radioresistance by preferential activation of the dna damage response. Nature 444: 756–60. 17051156 10.1038/nature05236

[9] van der Heide UA, Houweling AC, Groenendaal G, Beets-Tan RG, Lambin P (2012) Functional mri for radiotherapy dose painting. Magn Reson Imaging 30: 1216–23. 22770686 10.1016/j.mri.2012.04.010 PMC5134673

[10] Bentzen SM, Gregoire V (2011) Molecular imaging-based dose painting: a novel paradigm for radiation therapy prescription. Semin Radiat Oncol 21: 101–10. 21356478 10.1016/j.semradonc.2010.10.001 PMC3052283

[11] International Commission on Radiation Units & Measurements (ICRU) (1993) Prescribing, recording, and reporting photon beam therapy. In: International commission on radiation units and measurements, Bethesda MD, USA: ICRU report 50.

[12] ICRU (1999) Prescribing, recording, and reporting photon beam therapy (supplement to icru report 50). In: International commission on radiation units and measurements, Bethesda MD, USA: ICRU report 62.

[13] ICRU (2010) Prescribing, recording, and reporting imrt. In: International commission on radiation units and measurements, Washington DC, USA: ICRU report 83.

[14] Malinen E, Søvik A, Hristov D, Bruland ØS, Olsen DR (2006) Adapting radiotherapy to hypoxic tumours. Phys Med Biol 51: 4903–21. 16985278 10.1088/0031-9155/51/19/012

[15] Piccirillo SG, Combi R, Cajola L, Patrizi A, Redaelli S, et al (2009) Distinct pools of cancer stemlike cells coexist within human glioblastomas and display different tumorigenicity and independent genomic evolution. Oncogene 28: 1807–11. 19287454 10.1038/onc.2009.27

[16] Moore N, Lyle S (2011) Quiescent, slow-cycling stem cell populations in cancer: a review of the evidence and discussion of significance. J Oncol 2011. 10.1155/2011/396076 PMC2948913 20936110

[17] Vlashi E, Lagadec C, Vergnes L, Matsutani T, Masui K, et al (2011) Metabolic state of glioma stem cells and nontumorigenic cells. Proc Natl Acad Sci U S A 108: 16062–7. 21900605 10.1073/pnas.1106704108 PMC3179043

[18] Drasdo D, Höhme S (2005) A single-cell-based model of tumor growth in vitro: monolayers and spheroids. Phys Biol 2: 133–47. 16224119 10.1088/1478-3975/2/3/001

[19] Anderson RA, Chaplain MAJ, Rejniak K, editors (2007) In: Single-cell-based models in biology and medicine, Birkhauser-Verlag, Basel, Boston and Berlin.

[20] Fowler JF (1989) The linear-quadratic formula and progress in fractionated radiotherapy. Br J Radiol 62: 679–94. 2670032 10.1259/0007-1285-62-740-679

[21] Lee SP, Leu MY, Smathers JB, McBride WH, Parker RG, et al (1995) Biologically effective dose distribution based on the linear quadratic model and its clinical relevance. Int J Radiat Oncol Biol Phys 33: 375–89. 7673025 10.1016/0360-3016(95)00162-R

[22] Ramis-Conde I, Drasdo D, Anderson AR, Chaplain MA (2008) Modeling the inuence of the e-cadherin-beta-catenin pathway in cancer cell invasion: a multiscale approach. Biophys J 1: 155–165. 10.1529/biophysj.107.114678 PMC2426623 18339758

[23] Rietman EA, Friesen DE, Hahnfeldt P, Gatenby R, Hlatky L, et al.. (2013) An integrated multidisciplinary model describing initiation of cancer and the warburg hypothesis. Theor Biol Med Model 10. 10.1186/1742-4682-10-39 PMC3689044 23758735

[24] Gillies RJ, Verduzco D, Gatenby RA (2012) Evolutionary dynamics of carcinogenesis and why targeted therapy does not work. Nat Rev Cancer 12: 487–93. 22695393 10.1038/nrc3298 PMC4122506

[25] Preziosi L, Vitale G (2011) A multiphase model of tumor and tissue growth including cell adhesion and plastic reorganization. Math Models Methods Appl 21: 1901–32.

[26] Bellomo N, Delitala M (2008) From the mathematical kinetic, and stochastic game theory to modelling mutations, onset, progression and immune competition of cancer cells. Physics of Life Reviews 5: 183–206.

[27] Anderson AR, Quaranta V (2008) Integrative mathematical oncology. Nat Rev Cancer 8: 227–34. 18273038 10.1038/nrc2329

[28] Anderson AR, Weaver AM, Cummings PT, Quaranta V (2006) Tumor morphology and phenotypic evolution driven by selective pressure from the microenvironment. Cell 127: 905–15. 17129778 10.1016/j.cell.2006.09.042

[29] Agur Z, Vuk-Pavlović S (2012) Mathematical modeling in immunotherapy of cancer: personalizing clinical trials. Mol Ther 20: 1–2. 22215048 10.1038/mt.2011.272 PMC3255591

[30] Silva AS, Gatenby RA (2010) A theoretical quantitative model for evolution of cancer chemotherapy resistance. Biol Direct 5. 10.1186/1745-6150-5-25 PMC2868834 20406443

[31] Hoehme S, Brulport M, Bauer A, Bedawy E, Schormann W, et al (2010) Prediction and validation of cell alignment along microvessels as order principle to restore tissue architecture in liver regeneration. Proc Natl Acad Sci U S A 107: 10371–6. 20484673 10.1073/pnas.0909374107 PMC2890786

[32] Enderling H, Park D, Hlatky L, Hahnfeldt P (2009) The importance of spatial distribution of stemness and proliferation state in determining tumor radioresponse. Math Model Nat Phenom 4: 117–33.

[33] Kempf H, Bleicher M, Meyer-Hermann M (2010) Spatio-temporal cell dynamics in tumour spheroid irradiation. The European Physical Journal D 60: 177–93.

[34] Zacharaki EI, Stamatakos GS, Nikita KS, Uzunoglu NK (2004) Simulating growth dynamics and radiation response of avascular tumour spheroids-model validation in the case of an emt6/ro multicellular spheroid. Comput Methods Programs Biomed 76: 193–206. 15501506 10.1016/j.cmpb.2004.07.003

[35] Düchting W, Vogelsaenger T (1985) Recent progress in modelling and simulation of threedimensional tumor growth and treatment. Biosystems 18: 79–91. 3840706 10.1016/0303-2647(85)90061-9

[36] Enderling H, Anderson AR, Chaplain MA, Munro AJ, Vaidya JS (2006) Mathematical modeling of radiotherapy strategies for early breast cancer. J Theor Biol 241: 158–71. 16386275 10.1016/j.jtbi.2005.11.015

[37] Enderling H, Chaplain MAJ, Anderson ARA, Vaidya JS (2007) A mathematical model of breast cancer development, local treatment and recurrence. J Theor Biol 2: 245–259. 10.1016/j.jtbi.2006.12.010 17289081

[38] Enderling H, Chaplain MAJ, Hahnfeldt P (2010) Quantitative modeling of tumor dynamics and radiotherapy. Acta Biotheor 4: 341–353. 10.1007/s10441-010-9111-z 20658170

[39] Cappuccio A, Herrero MA, Núñez L (2009) Tumour radiotherapy and its mathematical modelling. Contemporary Mathematics 492: 77–102.

[40] O’Rourke SF, McAneney H, Hillen T (2009) Linear quadratic and tumour control probability modelling in external beam radiotherapy. J Math Biol 4–5: 799–817. 10.1007/s00285-008-0222-y 18825382

[41] Rockne R, Alvord ECJ, Rockhill JK, Swanson KR (2009) A mathematical model for brain tumor response to radiation therapy. J Math Biol 4–5: 561–578. 10.1007/s00285-008-0219-6 PMC3784027 18815786

[42] Rockne R, Rockhill JK, Mrugala M, Spence AM, Kalet I, et al (2010) Predicting the efficacy of radiotherapy in individual glioblastoma patients in vivo: a mathematical modeling approach. Phys Med Biol 12: 3271–3285. 10.1088/0031-9155/55/12/001 PMC3786554 20484781

[43] Gao X, McDonald JT, Hlatky L, Enderling H (2013) Acute and fractionated irradiation differentially modulate glioma stem cell division kinetics. Cancer Res 73: 1481–90. 23269274 10.1158/0008-5472.CAN-12-3429 PMC3594421

[44] Vainstein V, Kirnasovsky OU, Kogan Y, Agur Z (2012) Strategies for cancer stem cell elimination: insights from mathematical modeling. J Theor Biol 298: 32–41. 22210402 10.1016/j.jtbi.2011.12.016

[45] Dionysiou DD, Stamatakos GS, Gintides D, Uzunoglu N, Kyriaki K (2008) Critical parameters determining standard radiotherapy treatment outcome for glioblastoma multiforme: a computer simulation. Open Biomed Eng J 2: 43–51. 19662116 10.2174/1874120700802010043 PMC2701071

[46] Dahlberg WK, Azzam EI, Yu Y, Little JB (1999) Response of human tumor cells of varying radiosensitivity and radiocurability to fractionated irradiation. Cancer Res 59: 5365–9. 10537321

[47] Allam A, Taghian A, Gioioso D, Duffy M, Suit HD (1993) Intratumoral heterogeneity of malignant gliomas measured in vitro. Int J Radiat Oncol Biol Phys 27: 303–8. 8407404 10.1016/0360-3016(93)90241-m

[48] Stupp R, Hegi ME (2007) Targeting brain-tumor stem cells. Nat Biotechnol 25: 193–4. 17287755 10.1038/nbt0207-193

[49] Clarke MF, Dick JE, Dirks PB, Eaves CJ, Jamieson CH, et al (2006) Cancer stem cells–perspectives on current status and future directions: Aacr workshop on cancer stem cells. Cancer Res 66: 9339–44. 16990346 10.1158/0008-5472.CAN-06-3126

[50] Bonnet D, Dick JE (1997) Human acute myeloid leukemia is organized as a hierarchy that originates from a primitive hematopoietic cell. Nat Med 3: 730–7. 9212098 10.1038/nm0797-730

[51] Li L, Neaves WB (2006) Normal stem cells and cancer stem cells: the niche matters. Cancer Res 66: 4553–57. 16651403 10.1158/0008-5472.CAN-05-3986

[52] Dembinski JL, Krauss S (2009) Characterization and functional analysis of a slow cycling stem cell-like subpopulation in pancreas adenocarcinoma. Clin Exp Metastasis 26: 611–23. 19421880 10.1007/s10585-009-9260-0 PMC2776152

[53] Roesch A, Fukunaga-Kalabis M, Schmidt EC, Zabierowski SE, Brafford PA, et al (2010) A temporarily distinct subpopulation of slow-cycling melanoma cells is required for continuous tumor growth. Cell 141: 583–94. 20478252 10.1016/j.cell.2010.04.020 PMC2882693

[54] Moore N, Houghton J, Lyle S (2012) Slow-cycling therapy-resistant cancer cells. Stem Cells Dev 21: 1822–30. 21973238 10.1089/scd.2011.0477 PMC3376467

[55] Richichi C, Brescia P, Alberizzi V, Fornasari L, Pelicci G (2013) Marker-independent method for isolating slow-dividing cancer stem cells in human glioblastoma. Neoplasia 15: 840–7. 23814495 10.1593/neo.13662 PMC3689246

[56] Bansal A, Ramalingam S, Anant S (2013) Cancer stem cells in the origin and transformation of barrett’s esophagus: Current knowledge and areas of uncertainty. Immunogastroenterology 2: 9–21.

[57] Chen J, Li Y, Yu TS, McKay RM, Burns DK, et al (2012) A restricted cell population propagates glioblastoma growth after chemotherapy. Nature 488: 522–26. 22854781 10.1038/nature11287 PMC3427400

[58] Hegedüs B, Czirók A, Fazekas I, B’abel T, Madar’asz E, et al (2000) Locomotion and proliferation of glioblastoma cells in vitro: statistical evaluation of videomicroscopic observations. J Neurosurg 92: 428–34. 10701529 10.3171/jns.2000.92.3.0428

[59] Stamatakos GS, Antipas VP, Uzunoglu NK, Dale RG (2006) A four-dimensional computer simulation model of the in vivo response to radiotherapy of glioblastoma multiforme: studies on the effect of clonogenic cell density. Br J Radiol 79: 389–400. 16632619 10.1259/bjr/30604050

[60] Baumann M, Krause M, Hill R (2008) Exploring the role of cancer stem cells in radioresistance. Nat Rev Cancer 8: 545–54. 18511937 10.1038/nrc2419

[61] Enderling H, Hlatky L, Hahnfeldt P (2013) Cancer stem cells: A minor cancer subpopulation that redefines global cancer features. Front Oncol 3. 10.3389/fonc.2013.00076 PMC3625721 23596563

[62] Enderling H, Hlatky L, Hahnfeldt P (2009) Migration rules: tumours are conglomerates of selfmetastases. Br J Cancer 100: 1917–25. 19455139 10.1038/sj.bjc.6605071 PMC2714240

[63] Pine SR, Ryan BM, Varticovski L, Robles AI, Harris CC (2010) Microenvironmental modulation of asymmetric cell division in human lung cancer cells. Proc Natl Acad Sci U S A 107: 2195–200. 20080668 10.1073/pnas.0909390107 PMC2836660

[64] Knoblich JA (2010) Asymmetric cell division: recent developments and their implications for tumour biology. Nat Rev Mol Cell Biol 11: 849–60. 21102610 10.1038/nrm3010 PMC3941022

[65] Visvader JE, Lindeman GJ (2008) Cancer stem cells in solid tumours: accumulating evidence and unresolved questions. Nat Rev Cancer 8: 755–68. 18784658 10.1038/nrc2499

[66] Das S, Srikanth M, Kessler JA (2008) Cancer stem cells and glioma. Nat Clin Pract Neurol 4: 427–35. 18628751 10.1038/ncpneuro0862

[67] Barendsen GW, Bree CV, Franken NA (2001) Importance of cell proliferative state and potentially lethal damage repair on radiation effectiveness: implications for combined tumor treatments (review). Int J Oncol 19: 247–56. 11445835 10.3892/ijo.19.2.247

[68] Allalunis-Turner MJ, Barron GM 3rd, Day RS, Fulton DS, Urtasun RC (1992) Radiosensitivity testing of human primary brain tumor specimens. Int J Radiat Oncol Biol Phys 23: 339–43. 1316889 10.1016/0360-3016(92)90751-3

[69] Taghian A, Ramsay J, Allalunis-Turner MJ, Budach W, Gioioso D, et al (1993) Intrinsic radiation sensitivity may not be the major determinant of the poor clinical outcome of glioblastoma multiforme. Int J Radiat Oncol Biol Phys 25: 243–9. 8380568 10.1016/0360-3016(93)90345-v

[70] Araujo RP, McElwain DL (2004) A history of the study of solid tumour growth: the contribution of mathematical modelling. Bull Math Biol 66: 1039–91. 15294418 10.1016/j.bulm.2003.11.002

[71] Lowengrub JS, Frieboes HB, Jin F, Chuang YL, Li X, et al (2010) Nonlinear modelling of cancer: bridging the gap between cells and tumours. Nonlinearity 23: R1–R9. 20808719 10.1088/0951-7715/23/1/r01 PMC2929802

[72] Moreira J, Deutsch A (2002) Cellular automaton models of tumor development: a critical review. Advs Complex Syst 5: 1–21.

[73] Sottoriva A, Verhoeff JJ, Borovski T, McWeeney SK, Naumov L, et al (2010) Cancer stem cell tumor model reveals invasive morphology and increased phenotypical heterogeneity. Cancer Res 70: 46–56. 20048071 10.1158/0008-5472.CAN-09-3663

[74] Düchting W, Lehrig R, Rademacher G, Ulmer W (1989) Computer simulation of clinical irradiation schemes applied to in vitro tumor spheroids. Strahlenther Onkol 165: 873–8. 2603123

[75] Dormann S, Deutsch A (2002) Modeling of self-organized avascular tumor growth with a hybrid cellular automaton. In Silico Biol 2: 393–406. 12542422

[76] Ribba B, Marron K, Agur Z, Alarcón T, Maini PK (2005) A mathematical model of doxorubicin treatment efficacy for non-hodgkin’s lymphoma: investigation of the current protocol through theoretical modelling results. Bull Math Biol 67: 79–99. 15691540 10.1016/j.bulm.2004.06.007

[77] Maini PK, Gatenby RA (2006) Some mathematical modelling challenges and approaches in cancer. In: Nagl S, editor, Cancer Bioinformatics: From Therapy Design to Treatment, Chichester, UK: John Wiley and Sons, Ltd. 95–107. doi:10.1002/0470032898.ch5.

[78] Alfonso JCL, Buttazzo G, García-Archilla B, Herrero MA, Núñez L (2012) A class of optimization problems in radiotherapy dosimetry planning. Discr Cont Dyn Systems B 17: 1651–72.

[79] Cappuccio A, Herrero MA, Núñez L (2009) Biological optimization of tumor radiosurgery. Med Phys 36: 98–104. 19235378 10.1118/1.2986141

[80] Radszuweit M, Block M, Hengstler JG, Schöll E, Drasdo D (2009) Comparing the growth kinetics of cell populations in two and three dimensions. Phys Rev E Stat Nonlin Soft Matter Phys 79: 051907. 19518480 10.1103/PhysRevE.79.051907

[81] Block M, Schöll E, Drasdo D (2007) Classifying the growth kinetics and surface dynamics in growing cell populations. Phys Rev Lett 99: 248101–104. 18233492 10.1103/PhysRevLett.99.248101

[82] Cruchten SV, Broeck WVD (2002) Morphological and biochemical aspects of apoptosis, oncosis and necrosis. Anat Histol Embryol 31: 214–23. 12196263 10.1046/j.1439-0264.2002.00398.x

[83] Bursch W, Kleine L, Tenniswood M (1990) The biochemistry of cell death by apoptosis. Biochem Cell Biol 68: 1071–4. 2257115 10.1139/o90-160

[84] Goergen JL, Marc A, Engasser JM (1993) Determination of cell lysis and death kinetics in continuous hybridoma cultures from the measurement of lactate dehydrogenase release. Cytotechnology 11: 189–95. 7764124 10.1007/BF00749869

[85] Wells JE, Russell JB (1996) The effect of growth and starvation on the lysis of the ruminal cellulolytic bacterium fibrobacter succinogenes. Appl Environ Microbiol 62: 1342–6. 8919795 10.1128/aem.62.4.1342-1346.1996 PMC167900

[86] Gillespie DT (1977) Exact stochastic simulation of coupled chemical reactions. J Phys Chem 81: 2340–61.

[87] Bortz AB, Kalos MH, Lebowitz JL (1975) New algorithm for monte-carlo simulations of ising spin systems. J Comput Phys 17: 10–18.

[88] Fuller CD, Choi M, Forthuber B, Wang SJ, Rajagiriyil N, et al.. (2007) Standard fractionation intensity modulated radiation therapy (imrt) of primary and recurrent glioblastoma multiforme. Radiat Oncol 2. 10.1186/1748-717X-2-26 PMC1939706 17629934

[89] Narayana A, Yamada J, Berry S, Shah P, Hunt M, et al (2006) Intensity-modulated radiotherapy in high-grade gliomas: clinical and dosimetric results. Int J Radiat Oncol Biol Phys 64: 892–7. 16458777 10.1016/j.ijrobp.2005.05.067

[90] Stupp R, Tonn JC, Brada M, Pentheroudakis G (2010) Esmo guidelines working group. highgrade malignant glioma: Esmo clinical practice guidelines for diagnosis, treatment and follow-up. Ann Oncol 21. 10.1093/annonc/mdq187 20555079

[91] Korolev KS, Müller MJI, Karahan N, Murray AW, Hallatschek O, et al (2012) Selective sweeps in growing microbial colonies. Phys Biol 9: 026008. 22476106 10.1088/1478-3975/9/2/026008 PMC3359763

[92] Barendsen GW (1982) Dose fractionation, dose rate and iso-effect relationships for normal tissue responses. Int J Radiat Oncol Biol Phys 8: 1981–97. 6759484 10.1016/0360-3016(82)90459-x

[93] Emami B, Lyman J, Brown A, Coia L, Goiten M, et al (1991) Tolerance of normal tissue to therapeutic radiation. Int J Radiat Oncol Biol Phys 21: 109–22. 2032882 10.1016/0360-3016(91)90171-y

[94] Grimm J, LaCouture T, Croce R, Yeo I, Zhu Y, et al (2011) Dose tolerance limits and dose volume histogram evaluation for stereotactic body radiotherapy. J Appl Clin Med Phys 12: 267–92. 10.1120/jacmp.v12i2.3368 PMC5718687 21587185

[95] Dale R (2007) In: Radiobiological modelling in radiation oncology, London, UK: The British Institute of Radiology.

[96] Buatti JM, Marcus RB, Mendenhall WM, Friedman WA, Bova FJ (1996) Accelerated hyperfractionated radiotherapy for malignant gliomas. Int J Radiat Oncol Biol Phys 34: 785–92. 8598354 10.1016/0360-3016(95)02157-4

[97] Chaffer CL, Brueckmann I, Scheel C, Kaestli AJ, Wiggins PA, et al (2011) Normal and neoplastic nonstem cells can spontaneously convert to a stem-like state. Proc Natl Acad Sci U S A 108: 7950–5. 21498687 10.1073/pnas.1102454108 PMC3093533

[98] Tang DG (2012) Understanding cancer stem cell heterogeneity and plasticity. Cell Res 22: 457–72. 22357481 10.1038/cr.2012.13 PMC3292302

[99] Meacham CE, Morrison SJ (2013) Tumour heterogeneity and cancer cell plasticity. Nature 501: 328–37. 24048065 10.1038/nature12624 PMC4521623

[100] Lengauer C, Kinzler KW, Vogelstein B (1998) Genetic instabilities in human cancers. Nature 396: 643–9. 9872311 10.1038/25292

[101] Wang J, Guo LP, Chen LZ, Zeng YX, Lu SH (2007) Identification of cancer stem cell-like side population cells in human nasopharyngeal carcinoma cell line. Cancer Res 67: 3716–24. 17440084 10.1158/0008-5472.CAN-06-4343

[102] Gatenby RA (2009) A change of strategy in the war on cancer. Nature 459: 508–9. 19478766 10.1038/459508a

[103] Tixier F, Rest CCL, Hatt M, Albarghach N, Pradier O, et al (2011) Intratumor heterogeneity characterized by textural features on baseline 18f-fdg pet images predicts response to concomitant radiochemotherapy in esophageal cancer. J Nucl Med 52: 369–78. 21321270 10.2967/jnumed.110.082404 PMC3789272

