# Supplementary material for: Estimating Dose Painting Effects in Radiotherapy: A Mathematical Model
Source: PLoS One. 2014 Feb 26;9(2):e89380. doi: 10.1371/journal.pone.0089380 (PMC3935877; doi:10.1371/journal.pone.0089380)
Supplement: Document S1 — Details of computer simulations of the model of tumor growth and additional results. (PDF) [file pone.0089380.s001.pdf]

Estimating dose painting effects in radiotherapy: a mathematical model

Juan Carlos López Alfonso[1], **Nick Jagiella**[2,3], **Luis Núñez**[4], **Miguel A. Herrero**[1,*] and **Dirk Drasdo**[2,5,6]

[1] Department of Applied Mathematics, Faculty of Mathematics, Universidad Complutense de Madrid, Madrid, Spain.
[2] Institut National de Recherche en Informatique et en Automatique (INRIA), Domaine de Voluceau - Rocquencourt, Paris, France.
[3] Institute of Computational Biology, Helmholtz Center Munich, German Research Center for Environmental Health, Neuherberg, Germany.
[4] Radiophysics Department, Hospital Universitario Puerta de Hierro, Majadahonda, Madrid, Spain.
[5] University of Paris 6 (UPMC), CNRS UMR 7598, Laboratoire Jacques-Louis Lions, Paris, France.
[6] Interdisciplinary Center for Bioinformatics (IZBI), University of Leipzig, Leipzig, Germany.

∗ **E-mail:** herrero@mat.ucm.es

Supporting Information

1.- Details of computer simulations of the model of tumor growth and radiotherapy treatments.

1.1.- Cell processes and model parameters.

In this work a three-dimensional (3D) cellular automata (CA) model for tumor growth is developed, where each cell is considered as an individual agent (see [1], [2] and [3]). For computer simulations of the model, a three-dimensional Voronoi tessellation is implemented, where each lattice point will host only one cell at any time. The construction points of the Voronoi tessellation are defined by first generating a regular cubic lattice with lattice constant a, and then placing exactly one point at an arbitrary position within each cube of that lattice (resulting in an unstructured lattice with no rotational or translational symmetry, thus avoiding symmetry artifacts). Constant a is chosen such that the average volume of a Voronoi cell, $E[V] = a^3$, corresponds to that of a biological cell, $V = (\pi/6)d^3$. In this work the average cell diameter d will be assumed equal to $20\,\mu m$ (i.e. $a = (\pi/6)^{1/3}\, d = 16.12\,\mu m$). Thus, the domain of computer simulations is divided into $200 \times 200 \times 200$ lattice points, which corresponds to a total volume of about $33.51\,mm^3$. Actually, the modeling framework selected permits simulations to be scaled up to cubic centimeter sizes, though at the expense of lower spatial and functional resolution. Alternatively, hybrid models might be used, zooming in at the cell scale in regions of interest. However, we have chosen a CA model representing each cell individually to better understand the relation between tumor heterogeneity and simulated radiation treatment outcomes.

This model accounts for heterogeneous tumor growth. Specifically, two different tumor cell phenotypes (called, cancer cell (CC) and cancer stem cell (CSC)), which have markedly different biological and radiobiological properties, are allowed to coexist. The initial configuration of computer simulations is composed by 10^5 cells, out of which approximately 85% are CCs and 15% are CSCs. Tumor growth is kept track of until a size of about 10^6 cells is reached. At this time, the impact of different (homogeneous and heterogeneous) radiation therapies is simulated and discussed. In the case of tumor recurrence after radiation therapy, regrowth is allowed until a size of about 10^6 cells is attained, and then computer

simulations are stopped.

The cell processes considered in the model of tumor growth are summarized as follows:

○ **Cell Division (symmetric or asymmetric):** Cell replication on the lattice depends on the doubling time of the tumor cell phenotypes involved (τ). In the multi-cellular model the cell cycle is mimicked by a chain of Poisson processes. A Poisson process implies exponentially distributed waiting times. Moreover, a chain of m consecutive Poisson processes leads to an Erlang-distributed waiting time of the whole chain [4]. The integer parameter m determines the sharpness of the distribution around the average waiting time. This enables the model to capture a variety of different cell cycle time distribution shapes from exponentially distributed cell cycle times ($m = 1$) to very sharp Erlang-distributed cell cycle times ($m \to \infty$). In this case, the cell cycle is modeled as such a chain of 4 consecutive cell cycle phases ($G1$, S, $G2$ and M) which themselves are subdivided into a number of subprocesses (m_{G1}, m_S, m_{G2}, m_M). Each of the cell cycle phases has an average duration (τ_{G1}, τ_S, τ_{G2}, τ_M). This construction permits to control the average duration τ_p as well as the sharpness of the distribution of τ_p for each phase p individually (see Figure S1). Beginning in cycle step $i = 1$, a cell progresses in the cell cycle from i to $i + 1$ with rate m_p/τ_p, where p is the associated cell cycle phase. When $i = m_{G1} + m_S + m_{G2} + m_M$, the cell divides and both daughter cells reenter in the cell cycle with $i = 1$.

In our model of tumor growth, division of CCs is always supposed to be symmetric giving two identical daughter cells and with a limited replication capability. However, CSCs will be assumed to sustain either symmetric or asymmetric division, in which case one CSC and one CC will result from replication. Proliferation is only possible for cells located at a point having at least one free neighbor in the lattice (which is randomly chosen for replication). During cell division, the daughter cell is placed on this randomly selected free neighbor site. A cell with no free neighbors temporarily loses its ability to divide and becomes quiescent due to contact inhibition. The quiescence state is abandoned as soon as one of the surrounding lattice points becomes free. The duration of cell cycle for CCs is assumed to be significantly shorter than that of CSCs (see Table S1).

○ **Migration:** In this work, both tumor cell phenotypes considered are able to move to a randomly chosen free neighbor site with same (hopping) rate k_{mig}. We have considered two different migration rates, a comparatively low rate obtained from the cell diffusion constant (cf. for instance [1] and [5]) and a higher rate as estimated *in vitro* in [6] for a Glioblastoma Multiforme (GBM) cell line (see Table S1). The cell diffusion rate is estimated as $D = 6.5 \times 10^{-11}\ cm^2/s - 4.7 \times 10^{-12}\ cm^2/s$ (see Table of parameters in [1]). The diffusion coefficient depends on the properties of the cell and the surrounding media. Since $D = l^2\lambda$ ($\lambda =$ hopping rate $= k_{mig}$) and l the hopping distance (= cell diameter), then considering a cell diameter of $20\mu m$, we have that $\lambda = D/l^2 = D{\cdot}2.5{\times}10^5\ cm^2 = 2.5{\times}10^{-7}\ s^{-1} - 1.5{\times}10^{-5}\ s^{-1} = 0.009\ h^{-1} - 0.054\ h^{-1}$. Therefore, for the low migration case we have selected $k_{mig} = 0.025\ h^{-1}$, which is in the range estimated. On the other hand, for the high migration case, a $k_{mig} = 1.75\ h^{-1}$ has been selected. Similar estimates of this last migration rate have been independently derived for a GBM cell line in [6] and references therein.

○ **Death by radiation:** The effect of radiation on tumor cells (in terms of the corresponding surviving cell fraction) is estimated by means of the standard Linear-Quadratic (LQ) model (cf. [7] and [8]). We have considered that CSCs are more resistant to radiation than CCs. Moreover, for any of these two tumor cell phenotypes, quiescent cells are more radioresistant than proliferating ones. When a radiation dose is delivered on the tumor, four estimates on dying fractions (corresponding to proliferating / quiescent CC phenotype and to proliferating / quiescent CSC phenotype) for the total tumor volume or for the inner and outer tumor regions are computed and a corresponding amount of cells (randomly chosen) is then declared as being dead.

○ **Apoptosis (programmed cell death):** In our model, both tumor cell phenotypes considered are subject to apoptosis, a programmed cell death. When this process occurs, cells activate the apoptotic pathway, which will lead to cell shrinkage, nuclear fragmentation, chromatin condensation, chromosomal DNA fragmentation and cell fragmentation into apoptotic bodies. The corresponding process is accounted for in the model of tumor growth by changing from a proliferating or quiescent state to an apoptotic state (cell death) at a rate k_{apt} (see Table S1).

○ **Lysis:** Disposal of cellular debris resulting from apoptosis is carried out by a lysis process [9], for which a lysis rate $k_{lys} = 0.035\,h^{-1}$ (about $30\,h$) has been assumed (see Table S1). This rate is about 10-fold less than phagocytosis (digestion of cellular debris by macrophages) observed *in vivo* [10], but within the range reported for *in vitro* cultures (0.002 h^{-1} for *Hybridoma VO 208* cell line [11] to 0.07 h^{-1} for *Fibrobacter succinogenes* [12]). It should be noted in this context that tumor growth within the size limits considered in this work is closer to *in vitro* cultures than to *in vivo* situations. Lysis is mimicked in the model of tumor growth by means of a Poisson process, which removes dead cells from the lattice.

**Description**	**Symbol**	**Value/Range**	**Source**
Migration rate	k_{mig}	$0.025\,h^{-1}$ / $1.75\,h^{-1}$	[1], [6]
Apoptosis rate	k_{apt}	$4.17 \times 10^{-4}\,h^{-1}$	[13]
Lysis rate	k_{lys}	$0.035\,h^{-1}$	*(Assumed)*
Radiosensitivity (LQ model)	α	$0.48\,Gy^{-1}$	[14]
Radiosensitivity (LQ model)	β	$0.02\,Gy^{-2}$	[14]
Radiosensitivity (LQ model): Proliferating (CC)	ξ_{p1}	1.00	[15], [16]
Radiosensitivity (LQ model): Quiescent (CC)	ξ_{q1}	0.85	[15], [16]
Radiosensitivity (LQ model): Proliferating (CSC)	ξ_{p2}	0.30	[15], [16]
Radiosensitivity (LQ model): Quiescent (CSC)	ξ_{q2}	0.20	[15], [16]
CC cycle duration	τ_{cc}	$26h$	[17]
CSC cycle duration	τ_{csc}	$48h$, $72h$, $96h$	*(Assumed)*
Asymmetric division probability (CSC)	p_a	0.75, 0.50, 0.25	*(Assumed)*
Maximum number of cycle divisions (CC)	$---$	15	[13], [18]

Table S1: Model parameters used in computer simulations of tumor growth and radiotherapy treatments. Values for those parameters not found in the literature were assumed (see detailed explanation for lysis rate). In the remaining cases (asymmetric division probability and CSC cycle duration) some values were assumed, and the impact of different parameter sets on the resulting effects was subsequently analyzed.

1.2.- Time evolution of the system.

We have implemented a version of the Gillespie algorithm [19], adapted to the cell population system considered in this work. To this end, some explanations are in order. We shall index by μ a class of processes that cells can perform, say proliferation, migration, apoptosis and lysis. The related process rate, denoted by r_μ needs not be constant for all cells. For example, proliferating rates depend on the tumor cell phenotypes considered. Therefore, a process $P_{c,\mu}$ is specified by its process class μ, but also by the cell c where it takes place. For instance, if μ represents proliferation, the corresponding replication process can be summarized as follows:

$$P_{c,\mu} : c - [r_{c,\mu}] \rightarrow 2c$$

where $r_{c,\mu}$ is the rate at which the cell c implements the process μ (in this example, proliferation) and $2c$ represents the two daughter cells arising from c.

The algorithm describing the temporal evolution of the system is shortly described in the pseudo-code provided in Section 1.3 (see also Figure S1 for further details). Lines within braces at each step refer to the procedure sketched at the end of the description.

• **Step 0 (lines 1 to 10 - Initialization):** Read the initial set of cells (C) and the list of all possible processes at this stage of the system (P) from a data file (see lines 1 and 2). The initial tumor stage is composed by 10^5 cells, where about 85% are CCs and the remaining 15% are CSCs randomly distributed. Set the time variable t to zero and initialize the unit-interval uniform random number generator (URN) (see lines 3 and 4). When a size of about 10^6 cells is reached, a radiotherapy treatment is applied. Then the frequency and total number of sessions (r_{sf} and n_s), the dying cell fraction for each tumor cell phenotype (CC and CSC) after each session, and the respective cell states (proliferating and quiescent) are computed (see input parameters and lines 5 to 9). Moreover, the maximum number of cells allowed (mn_c) in computer simulations is also defined. On the other hand, the proportion of CSCs (p_{in}) to define the inner region for the case of high migration, when heterogeneous therapies are delivered, should be established (80% in our simulations). Notice that, for the case of low migration, p_{in} is equal to 100% which corresponds with the inner region where all CSCs are located.

• **Steps 1 to 5 (lines 11 to 79 - Time evolution):** A step-by-step time evolution is processed within the global loop. The following Steps 1 to 5 are repeated until the system reaches the end time of simulation t_{max}, there are no more processes to execute (for instance, all cells are dead) or the maximum number of cells considered is reached ($mn_c = 10^6$) at the end of the recurrence tumor stage after radiation therapy.

• **Step 1 (line 12 - Rates):** Calculate and store as r_Σ the sum of rates $r_{c,\mu}$ of all processes $P_{c,\mu} \in P$.

• **Step 2 (lines 13 and 14 - Time increment):** Generate a random number u_1 using the unit-interval uniform random number generator (URN), and calculate the time increment $\tau = -\frac{\ln(u_1)}{r_\Sigma}$.

• **Step 3 (lines 15 to 23 - Process selection):** The process $P_{b,v}$ to perform during this iteration is chosen randomly from the list of all processes P taking into account that the probability of each process $P_{c,\mu} \in P$ to be chosen is proportional to its rate $r_{c,\mu}$. As proposed by Gillespie in [19], this can be done as follows: generate a second random number u_2 using the unit-interval uniform random number generator (URN). Then step-by-step sum up as $r_\Sigma^{'}$ the rates $r_{c,\mu}$ of all processes $P_{c,\mu} \in P$ until the condition $r_\Sigma^{'} - r_{c,\mu} < u_2 \cdot r_\Sigma \leq r_\Sigma^{'}$ is satisfied. Then store $P_{c,\mu}$ as the selected process in $P_{b,v}$.

• **Step 4 (lines 24 and 25 - Update):** Increase the time t by τ, and adjust the cell set to account for what has happened during process $P_{b,v}$ (e.g. remove cells, add cells, move cells and/or change environment of a cell). Update the list of possible processes according to the new cell set (e.g. add/remove processes after an environmental change).

• **Step 5 (lines 26 to 78 - Radiation therapy):** Radiation therapy starts when the tumor reaches a size greater than or equal to mn_c. Radiation is delivered over the time corresponding with the number and frequency of sessions selected. Notice that in the case of weekend interruptions the variable rs_f should be incremented in 72 hours after weekly consecutive sessions, and afterwards this period will be changed back to the initial frequency of treatment sessions. For homogeneous therapies a number of cells is randomly selected on the whole tumor corresponding with the dying cell fraction of proliferating and

quiescent states for CCs and CSCs respectively. However, for heterogeneous therapies this selection is separately made within the inner sphere and in the rest of the tumor, and the dying cell fractions are computed for the corresponding radiation dose delivered on each region. At the end of each session, the cell state of this set of cells is updated to dead. Alternatively, one might introduce the death process by radiation as a rate. However, we assume that this process is very fast, in which case the algorithm we chose is much more efficient.

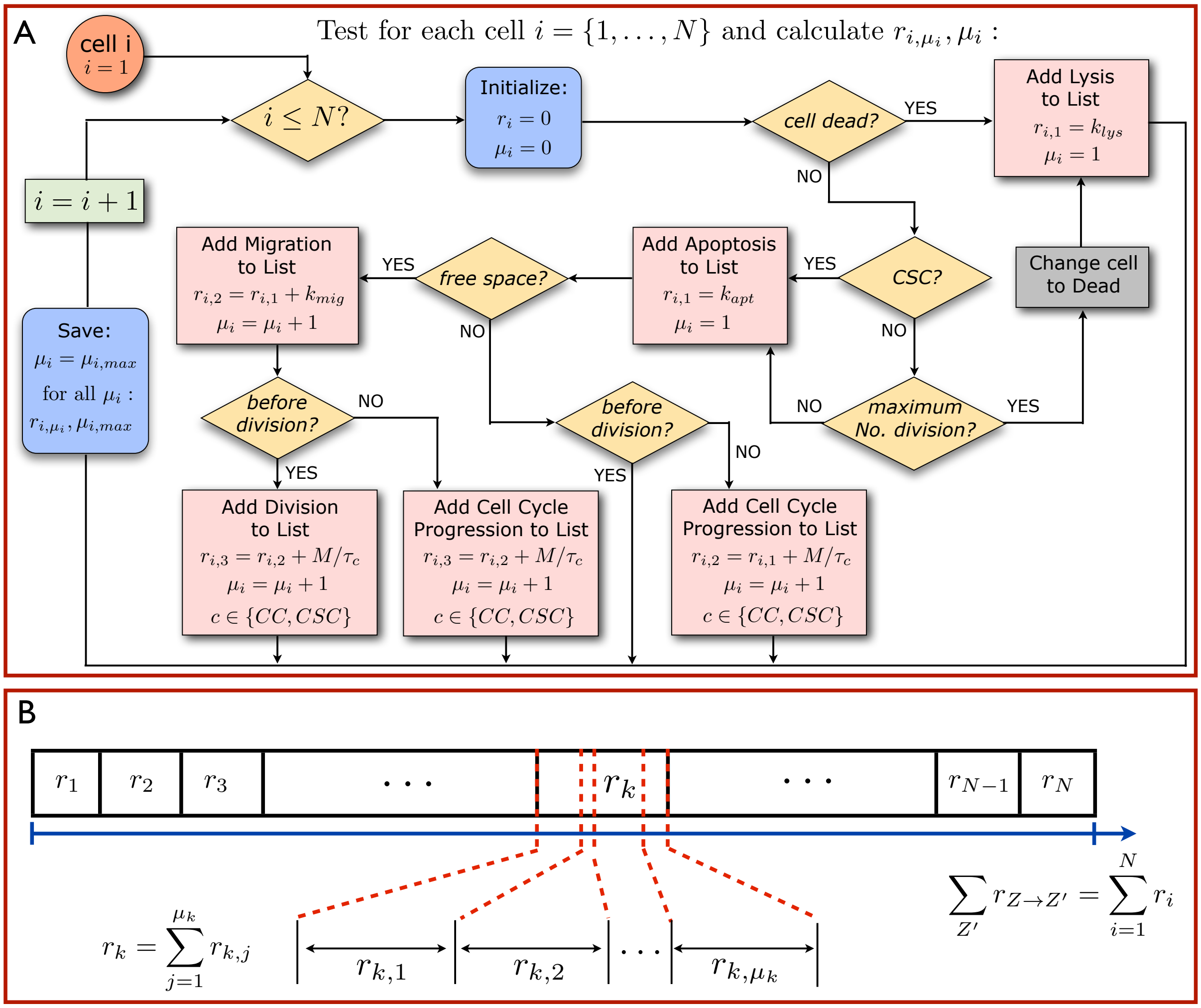

Figure S1: The flowchart of a cell in the model of tumor growth. (A) The flowchart illustrates how the accessible states and rates for the respective transition for a configuration of N cells, both CCs and CSCs, are determined algorithmically in the computer program. Here, $M = m_{G_1} + m_S + m_{G_2} + m_M$. The possible transitions are investigated cell by cell. For each cell ($i = 1, \ldots, N$) all processes that cell i can perform are determined and saved in a list. If cell i is dead, it can only undergo lysis with rate k_{lys}. Accordingly, the rate $r_{i,1}$ with which cell i can change its state becomes equal to the lysis rate. Notice that this includes cells of tumor phenotype CC which reached its maximum number of cell cycles and consequently die. If cell i is alive, apoptosis is added as every cell can undergo apoptosis in the model, so that $r_{i,1} = k_{apt}$. If in addition a lattice site is free next to the cell, it may migrate, so $r_{i,2} = r_{i,1} + k_{mig}$. In this way the total $r_{i,\mu}$ of cell i is computed, and all intermediate values, ($r_{i,1}$, $r_{i,2}$, ...) are saved. The total rate at which the current cell configuration changes is then given by $r_{tot} = \sum_{i=0}^{N} r_{i,\mu_{i,max}}$. (B) To select a process, a random number ξ is chosen in $[0, r_{tot})$, and that process p of cell k is performed, for which either $\sum_{i=1}^{k-1} r_{i,\mu_{i,max}} + r_{k,p} \leq \xi < \sum_{i=1}^{k-1} r_{i,\mu_{i,max}} + r_{k,p+1}$, or $p = \mu_{k,max}$ and $\sum_{i=1}^{k} r_{i,\mu_{i,max}} \leq \xi < \sum_{i=1}^{k} r_{i,\mu_{i,max}} + r_{k+1,1}$. Concerning radiation effects, cells are picked randomly and killed according to the corresponding surviving cell fraction estimate.

1.3.- Pseudo-Code Guidelines.

Input parameters:

t_{max} := maximum duration of simulations
rs_f := frequency of radiation sessions
n_s := total number of radiation sessions
mn_c := maximum number of cells allowed in simulations
p_{in} := proportion of CSCs to define the inner region (for heterogeneous therapies with the high migration rate)

Initialization:

1 – Input the initial set of cells $C := \{c \mid c \text{ is a cell within the volume } V \text{ where } c \in \{CC, CSC\}\}$
2 – Determine the processes array $P := \{P_{c,\mu} \mid \text{if } c \in C \text{ and } c \text{ is reactant of } \mu\}$
3 – Set $t := 0$
4 – Initialize URN
5 – Initialize the dying cell fraction for each tumor cell phenotype (CC and CSC) and cell state (proliferating and quiescent), where D is the radiation dose for homogeneous therapies:

6 – Set $p_{CC} := \left(1 - e^{-\xi_{p1}\left(\alpha D + \beta D^2\right)}\right)$
7 – Set $q_{CC} := \left(1 - e^{-\xi_{q1}\left(\alpha D + \beta D^2\right)}\right)$
8 – Set $p_{CSC} := \left(1 - e^{-\xi_{p2}\left(\alpha D + \beta D^2\right)}\right)$
9 – Set $q_{CSC} := \left(1 - e^{-\xi_{q2}\left(\alpha D + \beta D^2\right)}\right)$

# Notice that in the case of heterogeneous therapies, D_{in} and D_{out} should be defined with the corresponding dying cell fractions for each tumor cell phenotype (CC and CSC) and cell state (proliferating and quiescent) respectively.

10 – Define r_{rad}, s_{rad}, d_s and d_r (auxiliary variables):
Set r_{rad} := *false* # *conditional: start irradiation*
Set s_{rad} := *false* # *conditional: stop irradiation*
Set d_s := 0 # *delivered number of treatment sessions*
Set d_r := 0 # *radius partitions to define the inner region (for heterogeneous therapies with the high migration rate)*

11 – **REPEAT**

Sum over all process rates:
12 – Calculate $r_\Sigma := \sum_{P_{c,\mu} \in P} r_{c,\mu}$

Calculate time step τ:
13 – Generate u_1 from URN
14 – Take $\tau := -\frac{\ln(u_1)}{r_\Sigma}$

Select process $P_{b,v}$ regarding to its probability $P_{b,v} = \frac{r_{b,v}}{r_\Sigma}$:
15 – Generate u_2 from URN
16 – Set $r'_\Sigma := 0$

17 – for all $P_{c,\mu} \in P$ do
18 – if $(r'_\Sigma < u_2 \cdot r_\Sigma \leq r'_\Sigma + r_{c,\mu})$ then
Select cell b and process class v:

$b := c$
$v := \mu$
end if
Put $r'_{\Sigma} := r'_{\Sigma} + r_{c,\mu}$
end for

Execute process $P_{b,v}$:
Put $t := t + \tau$
Adjust C and P according to $P_{b,v}$

Applying radiation therapy:
if ((length(C) $\geq mn_c$) or ($r_{rad} == \mathit{true}$) and ($s_{rad} == \mathit{false}$)) then
if (($d_s == 0$) or (($t - t_s$) $== rs_f$))
(taking into account weekend interruptions if it is the case) then
if ($d_s == 0$) then
Compute the center-of-mass:
$m_x := \frac{\sum_{c \in C} x_i}{\text{length}(C)}$; $m_y := \frac{\sum_{c \in C} y_i}{\text{length}(C)}$; $m_z := \frac{\sum_{c \in C} z_i}{\text{length}(C)}$
Compute the diameter of tumor regions:
Set $r_{out} := 0$
Set $r_{in} := 0$

for all $c \in C$ do
$c_d := \sqrt{(c_x - m_x)^2 + (c_y - m_y)^2 + (c_z - m_z)^2}$
if ($c_d > r_{out}$) then
$r_{out} := c_d$
end if
end for
if (Heterogeneous therapy) then
N_{CSC} := Compute the number of CSCs in C
if (Low migration rate) then
for all $c \in C$ and c is CSC do
$c_d := \sqrt{(c_x - m_x)^2 + (c_y - m_y)^2 + (c_z - m_z)^2}$
if ($c_d > r_{in}$) then
$r_{in} := c_d$
end if
end for
else # *High migration rate*
for ($i = d_r$; $i > 0$; $i - -$) do
Set $c_{in} := 0$
for all $c \in C$ and c is CSC do
$c_d := \sqrt{(c_x - m_x)^2 + (c_y - m_y)^2 + (c_z - m_z)^2}$
if ($c_d \leq (r_{out}/d_r) * i$) then
$c_{in} := c_{in} + 1$
end if
end for
if ($c_{in} \geq p_{in} * N_{CSC}$) then
$r_{in} := (r_{out}/d_r) * i$
break
end if
end for

61 – end if
62 – end if
63 – end if

For homogeneous therapies select randomly on the whole tumor:

64 – A number of proliferating CCs corresponding to:
p_{CC}
65 – A number of quiescent CCs corresponding to:
q_{CC}
66 – A number of proliferating CSCs corresponding to:
p_{CSC}
67 – A number of quiescent CSCs corresponding to:
q_{CSC}

# Notice that for heterogeneous therapies this selection is separately made within the inner sphere (center-of-mass to r_{in}) and in the rest of the tumor (r_{in} to r_{out}) for the corresponding radiation dose delivered on each region (D_{in} and D_{out}) respectively.

68 – Change cell state of the selected set of cells to dead
69 – Adjust P for all selected cells

70 – $d_s := d_s + 1$
71 – $r_{rad} := true$

72 – if ($d_s == n_s$) then
73 – $r_{rad} := false$
74 – $s_{rad} := true$
75 – end if

76 – $t_s := t$ *# update time of the last delivered treatment session*
77 – end if
78 – end if
79 – **UNTIL** ($t \geq t_{max}$) or ($P = \varnothing$) or ((length(C) $\geq mn_c$) and ($s_{rad} == true$))

2.- Additional results: radiotherapy treatments and number of CSCs at the pre-treatment and recurrence tumor stages.

In this section, additional results for heterogeneous and homogeneous radiation therapies considered in this work are presented and other model properties are investigated.

2.1.- Heterogeneous vs. homogeneous therapies for the low and high migration cases.

To begin with, in Tables S2 and S3 (to be compared to Tables 4 and 5 in the article respectively) the number of CSCs at the recurrence tumor stage (with the corresponding standard deviation) for heterogeneous and the corresponding averaged homogeneous radiation therapies considered in the article are provided. Moreover, heterogeneous and homogeneous radiation therapies required to achieve tumor control are also shown. This is done for the values of p_a (0.75, 0.50 and 0.25), CSC cycle duration ($96h$, $72h$ and $48h$) and migration rate k_{mig} ($0.025h^{-1}$, $1.75h^{-1}$) considered in Table S1.

		Heterogeneous therapy		Homogeneous therapy	
p_a	τ_{csc}	**No Control**	**Control**	**No Control**	**Control**
**0.75**	**96h**	$---$	$2.0Gy - 2.5Gy^{(1)}$	$2.10Gy^{(1)}$	$2.5Gy$
				(83/7.87)	
	**72h**	$---$	$2.0Gy - 2.5Gy^{(2)}$	$2.10Gy^{(2)}$	$2.5Gy$
				(264/14.82)	
	**48h**	$2.0Gy - 2.5Gy^{(3)}$	$2.0Gy - 2.7Gy^{(4)}$	$2.10Gy^{(3)}/2.12Gy^{(4)}$	$2.7Gy$
		(107/8.53)		(603/41.22)/(514/13.65)	
**0.50**	**96h**	$---$	$2.0Gy - 2.9Gy^{(5)}$	$2.15Gy^{(5)}$	$2.9Gy$
				(814/37.57)	
	**72h**	$---$	$2.0Gy - 2.9Gy^{(6)}$	$2.17Gy^{(6)}$	$2.9Gy$
				(2130/86.68)	
	**48h**	$2.0Gy - 2.9Gy^{(7)}$	$2.0Gy - 3.4Gy^{(8)}$	$2.19Gy^{(7)}/2.30Gy^{(8)}$	$3.4Gy$
		(1785/78.31)		(16757/243.46)/(12208/456.08)	
**0.25**	**96h**	$---$	$2.0Gy - 3.3Gy^{(9)}$	$2.23Gy^{(9)}$	$3.3Gy$
				(3961/171.88)	
	**72h**	$---$	$2.0Gy - 3.3Gy^{(10)}$	$2.27Gy^{(10)}$	$3.3Gy$
				(14495/274.86)	
	**48h**	$2.0Gy - 3.3Gy^{(11)}$	$2.0Gy - 3.9Gy^{(12)}$	$2.32Gy^{(11)}/2.47Gy^{(12)}$	$3.9Gy$
		(4457/232.67)		(113546/1393.2)/(96346/1141.3)	

Table S2: Classification of heterogeneous and homogeneous radiation therapies for the low migration case. In all cases, treatment sessions were scheduled along 6 weeks separated by 24 hours intervals except for weekends, where a 72 hours interval is allowed. Data corresponding to 20 simulations (with different seeds of a random number generator) are presented. In the heterogeneous therapies, radiation doses are specified both for the outer (left) and inner (right) tumor regions, each case being indexed from (1) to (12). The averaged dose for any of the previous cases is labeled with the same number in the columns corresponding to homogeneous therapies. Within braces, the number of CSCs at the recurrence tumor stage and the corresponding standard deviation are also provided. See Figure 9 (A) in the article where some of these results are shown.

		Heterogeneous therapy		Homogeneous therapy	
p_a	τ_{csc}	No Control	Control	No Control	Control
**0.75**	**96h**	$---$	$2.0Gy - 2.5Gy^{(1)}$	$2.10Gy^{(1)}$	$2.5Gy$
				(135/11.88)	
	**72h**	$---$	$2.0Gy - 2.5Gy^{(2)}$	$2.11Gy^{(2)}$	$2.5Gy$
				(216/9.24)	
	**48h**	$2.0Gy - 2.5Gy^{(3)}$	$2.2Gy - 2.7Gy^{(4)}$	$2.13Gy^{(3)}/2.33Gy^{(4)}$	$2.7Gy$
		(294/16.73)		(536/37.21)/(267/10.61)	
**0.50**	**96h**	$2.0Gy - 2.9Gy^{(5)}$	$2.3Gy - 2.9Gy^{(6)}$	$2.23Gy^{(5)}/2.45Gy^{(6)}$	$2.9Gy$
		(349/21.89)		(634/43.73)/(251/13.86)	
	**72h**	$2.0Gy - 2.9Gy^{(7)}$	$2.6Gy - 2.9Gy^{(8)}$	$2.25Gy^{(7)}/2.70Gy^{(8)}$	$2.9Gy$
		(815/36.70)		(1579/85.56)/(466/29.65)	
	**48h**	$2.0Gy - 2.9Gy^{(9)}$	$2.8Gy - 3.4Gy^{(10)}$	$2.29Gy^{(9)}/3.00Gy^{(10)}$	$3.4Gy$
		(12712/455.01)		(19344/447.66)/(1582/48.38)	
**0.25**	**96h**	$2.0Gy - 3.3Gy^{(11)}$	$2.4Gy - 3.3Gy^{(12)}$	$2.36Gy^{(11)}/2.65Gy^{(12)}$	$3.3Gy$
		(1448/71.75)		(2419/160.03)/(769/59.82)	
	**72h**	$2.0Gy - 3.3Gy^{(13)}$	$2.7Gy - 3.3Gy^{(14)}$	$2.43Gy^{(13)}/2.90Gy^{(14)}$	$3.3Gy$
		(2646/136.52)		(11318/335.88)/(2020/77.78)	
	**48h**	$2.0Gy - 3.3Gy^{(15)}$	$3.4Gy - 3.9Gy^{(16)}$	$2.52Gy^{(15)}/3.60Gy^{(16)}$	$3.9Gy$
		(78073/3054.90)		(191730/3747.03)/(7716/174.66)	

Table S3: Classification of heterogeneous and homogeneous radiation therapies for the high migration case. In all cases, treatment sessions were scheduled along 6 weeks separated by 24 hours intervals except for weekends, where a 72 hours interval is allowed. Data corresponding to 20 simulations (with different seeds of a random number generator) are presented. In the heterogeneous therapies, radiation doses are specified both for the outer (left) and inner (right) tumor regions, each case being indexed from (1) to (16). The averaged dose for any of the previous cases is labeled with the same number in the columns corresponding to homogeneous therapies. Within braces, the number of CSCs at the recurrence tumor stage and the corresponding standard deviation are also provided. See Figure 9 (B) in the article where some of these results are shown.

2.2.- Consequences of assuming that CSCs cycle faster than CCs.

So far we have kept to the standard assumption that the duration of cell cycle for CSCs is significantly longer than that of CCs (cf. [20], [21], [22], [23], [24], [25], [26], [27], [28] and [29]). For completeness, we next shortly describe the results provided by our model of tumor growth under the opposite situation that CSCs cycle faster than CCs. To do that, we have considered the cases where CSC cycle lasts 26 hours (respectively 18 hours) which is equal to (respectively less than) the 26 hours cell cycle selected for CCs. In Tables S4 and S5 (see also Figures S2, S3 and S4) we provide estimates of the tumor inner region diameter and number of CSCs before radiation treatment starts, both for the low and high migration cases and values of p_a equal to 0.75, 0.50 and 0.25 respectively.

As expected, in these cases the inner core where most CSCs remain concentrated is now larger than when slow-cycling CSCs is assumed, see Tables S4 and S5 (to be compared to Tables 1 and 2 in the article). In particular, we recall that in the slow-cycling CSCs case for the low migration case such inner regions, where 100% of CSCs are located, ranged from 15% to 25% of the total tumor volume. For the case of high migration the volume where at least 80% of CSCs are located ranged from 21% to 40% of the volume where 90% of total cells (CCs and CSCs) are located. Notice that, as reported in the article, the average diameter of the tumor for the low migration case is about $2680\mu m$, while for the high migration

case the average diameter of the region where 90% of total cells (CCs and CSCs) are located is about $3120\mu m$ (with standard deviations of $56\mu m$ and $186\mu m$ over 20 simulation performed for each parameter set considered respectively).

When the assumption that CSCs cycle faster than CCs is made, we obtain that the values of the average diameter of the tumor and the region where the 90% of total cells (CCs and CSCs) are located for the low and high migration cases respectively, are exactly those recalled above. This is due to the fact that computer simulations are executed for the same maximum number of cells (10^6) keeping the same migration rates. However, in the case of low migration now considered and CSC cycle duration equal to $26h$ (respectively $18h$) tumor inner regions range from 20% to 83% (respectively 22% to 100%) of the total tumor volume (see Table S4, and Figures S2 (A) and S3 (A)). For the case of high migration and CSC cycle duration equal to $26h$ (respectively $18h$) these inner regions further expand, so that the volume where 80% of CSCs remain confined ranges now from 28% to 44% (respectively 33% to 51%) of the volume where 90% of total cells (CCs and CSCs) are located (see Table S5, and Figures S2 (B) and S3 (B)).

	$p_a = 0.75$		$p_a = 0.50$		$p_a = 0.25$	
τ_{csc}	**Diameter**	**CSCs**	**Diameter**	**CSCs**	**Diameter**	**CSCs**
**26h**	1567.3 μm	18217	1826.2 μm	21417	2516.3 μm	29873
	[57.16 μm]	[71.82]	[78.22 μm]	[145.66]	[37.87 μm]	[765.80]
**18h**	1623.5 μm	19151	2412.9 μm	27846	2678.3 μm	84134
	[63.48 μm]	[75.48]	[67.77 μm]	[573.03]	[48.75 μm]	[3104.15]

Table S4: Estimates of the tumor inner region diameter and number of CSCs before irradiation for the low migration case and fast-cycling CSCs. Diameter is that of an inner sphere where 100% of CSCs are located. CSCs number is computed before radiation therapy treatment starts. Within brackets the corresponding standard deviations are also provided. Data corresponding to 20 simulations (with different seeds of a random number generator) for each case considered. See also Figure S2 (A), S3 (A) and S4 (A) where these results are represented.

	$p_a = 0.75$		$p_a = 0.50$		$p_a = 0.25$	
τ_{csc}	**Diameter**	**CSCs**	**Diameter**	**CSCs**	**Diameter**	**CSCs**
**26h**	2048.1 μm	23084	2195.0 μm	38081	2365.7 μm	64786
	[72.81 μm]	[485.90]	[84.28 μm]	[1227.08]	[69.15 μm]	[2086.43]
**18h**	2156.5 μm	25791	2284.4 μm	54628	2495.4 μm	124584
	[62.38 μm]	[584.35]	[93.49 μm]	[1502.97]	[71.36 μm]	[2339.59]

Table S5: Estimates of the tumor inner region diameter and number of CSCs before irradiation for the high migration case and fast-cycling CSCs. Diameter is that of an inner sphere where 80% of CSCs are located. CSCs number is computed before radiation therapy treatment starts. Within brackets the corresponding standard deviations are also provided. Data corresponding to 20 simulations (with different seeds of a random number generator) for each case considered. See also Figure S2 (B), S3 (B) and S4 (B) where these results are represented.

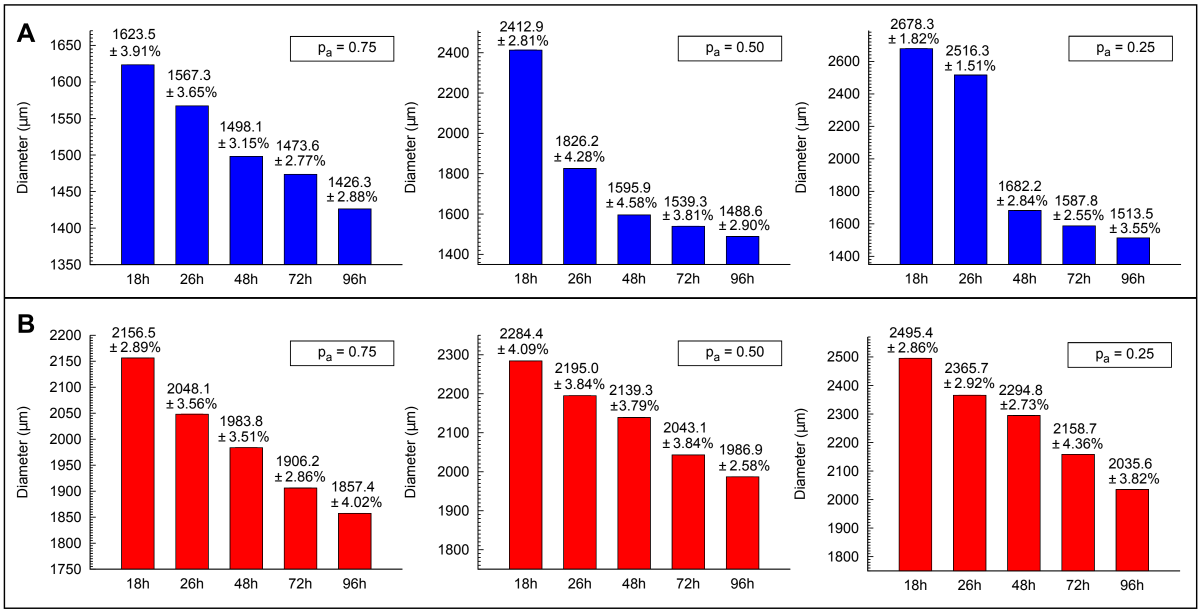

Figure S2: Representation of the tumor inner region diameter for the low and high migration cases. Diameters of the tumor inner region at the pre-treatment stage (where about 10^6 cells are present) and the corresponding standard deviations after performing 20 simulations in each case (with different seeds of a random number generator) are shown. Results are provided for the cases $p_a = 0.75$, $p_a = 0.50$ and $p_a = 0.25$ (left, middle, right) and CSC cycle durations equal to $18h$, $26h$, $48h$, $72h$ and $96h$. (A) Diameters of the tumor inner sphere where 100% of CSCs are located for the low migration case. (B) Diameters of the tumor inner sphere where 80% of CSCs are located for the high migration case. See Tables 2 and 3 in the article, and Tables S4 and S5 above.

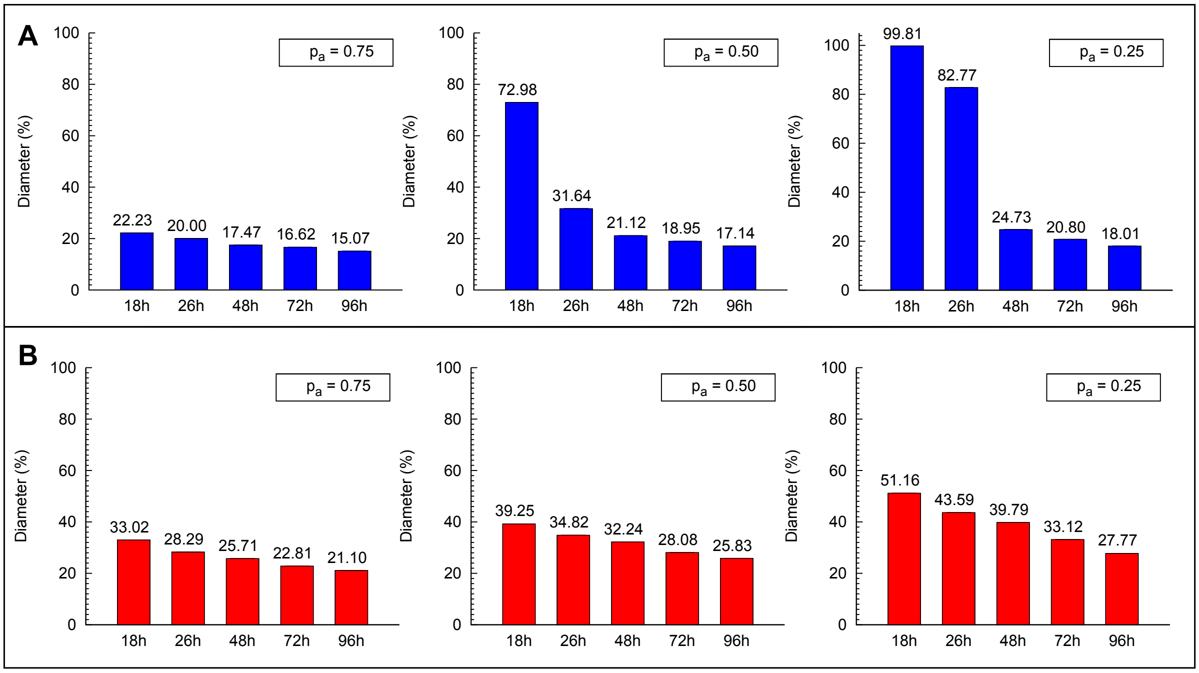

Figure S3: Representation of the volume proportion of the inner region respect to the volume of the outer region for the low and high migration cases. Volume of the tumor inner region at the pre-treatment stage (where about 10^6 cells are present) and the corresponding standard deviations after performing 20 simulations in each case (with different seeds of a random number generator) are shown. Results are provided for the cases $p_a = 0.75$, $p_a = 0.50$ and $p_a = 0.25$ (left, middle, right) and CSC cycle durations equal to $18h$, $26h$, $48h$, $72h$ and $96h$. (A) Volume proportion of the tumor inner sphere where 100% of CSCs are located for the low migration case with respect to the total tumor volume ($2680\mu m$). (B) Volume proportion of the tumor inner sphere where 80% of CSCs are located for the high migration case with respect to the region where 90% of total cells (CCs and CSCs) are located ($3120\mu m$). See Tables 2 and 3 in the article, and Tables S4 and S5 above.

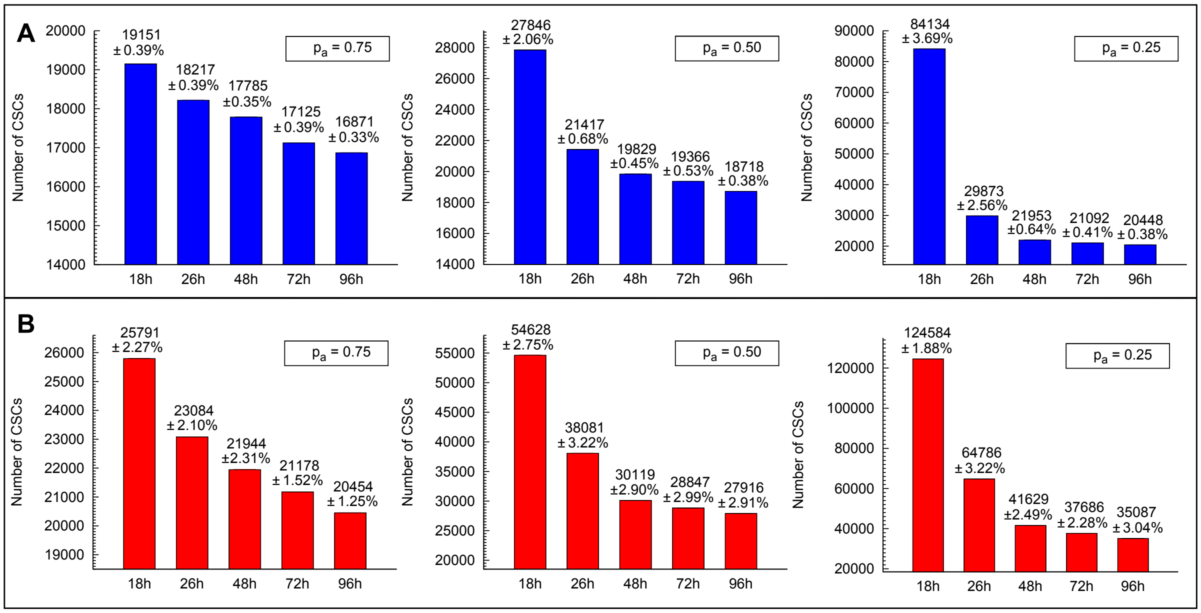

Figure S4: Representation of the number of CSCs before irradiation for the low and high migration cases. Number of CSCs at the pre-treatment stage (where about 10^6 cells are present) and the corresponding standard deviations after performing 20 simulations in each case (with different seeds of a random number generator) are shown. Results are provided for the cases $p_a = 0.75$, $p_a = 0.50$ and $p_a = 0.25$ (left, middle, right), CSC cycle durations equal to $18h$, $26h$, $48h$, $72h$ and $96h$, for the low (A) and high (B) migration cases. See Tables 2 and 3 in the article, and Tables S4 and S5 above.

To complete our current discussion of the fast-cycling CSCs situation, we next provide in Tables S6 and S7 (see also Figures S5 and S6) some results concerning the performance of heterogeneous and the corresponding averaged homogeneous radiation therapies for CSC cycle durations equal to $26h$ and $18h$ respectively. Moreover, the number of CSCs at the recurrence tumor stages and radiation doses for heterogeneous and homogeneous therapies needed to obtain tumor control are also shown.

As it turns out, as CSC cycle duration and p_a decrease, the standard fractionation (30 sessions delivered 5 days a week at 24 hours intervals with weekend interruptions) is not enough to treat the resulting tumors with clinically acceptable radiation doses. In both cases, heterogeneous and homogeneous radiation therapies and for some model parameter sets a fractionation protocol delivered in 7 days a week along 30 sessions at 24 hours intervals is advisable instead. Moreover, in the worst scenario, which corresponds to CSC cycle duration equal to $18h$ and $p_a = 0.25$, the total radiation dose needed to achieve tumor control with any of these fractionation protocols is too high to be considered as a treatment option. However, an inspection of Tables S6 and S7 (see also Figures S5 and S6) reveals that for all model parameter sets considered, heterogeneous radiation therapies yield better results than their averaged homogeneous counterparts.

		Heterogeneous therapy		Homogeneous therapy	
p_a	τ_{csc}	**No Control**	**Control**	**No Control**	**Control**
**0.75**	**26h**	$2.0Gy - 2.5Gy^{(1)}$	$2.0Gy - 3.2Gy^{(2)}$	$2.10Gy^{(1)}/2.24Gy^{(2)}$	$3.2Gy$
		$[63Gy]^{\dagger}$	$[67.2Gy]^{\dagger}$	$[63Gy]^{\dagger}/[67.2Gy]^{\dagger}$	$[96Gy]^{\dagger}$
		(3085/116.59)		(11904/232.85)/(7913/224.15)	
	**18h**	$2.0Gy - 2.5Gy^{(3)}$	$2.0Gy - 3.9Gy^{(4)}$	$2.11Gy^{(3)}/2.42Gy^{(4)}$	$3.9Gy$
		$[63.3Gy]^{\dagger}$	$[72.6Gy]^{\dagger}$	$[63.3Gy]^{\dagger}/[72.6Gy]^{\dagger}$	$[117Gy]^{\dagger}$
		(26579/586.17)		(57890/951.47)/(34675/854.97)	
**0.50**	**26h**	$2.0Gy - 2.9Gy^{(5)}$	$2.0Gy - 4.3Gy^{(6)}$	$2.28Gy^{(5)}/2.73Gy^{(6)}$	$4.3Gy$
		$[68.4Gy]^{\dagger}$	$[81.9Gy]^{\dagger}$	$[68.4Gy]^{\dagger}/[81.9Gy]^{\dagger}$	$[129Gy]^{\dagger}$
		(96626/1776.42)		(182051/3485.87)/(123683/2601.55)	
	**18h**	$2.0Gy - 2.9Gy^{(7)}$	$2.0Gy - 4.9Gy^{(8)}$	$2.66Gy^{(7)}/4.12Gy^{(8)}$	$4.9Gy$
		$[79.8Gy]^{\ddagger}$	$[123.6Gy]^{\ddagger}$	$[79.8Gy]^{\ddagger}/[123.6Gy]^{\ddagger}$	$[147Gy]^{\ddagger}$
		(241936/7613.76)		(357911/9430.74)/(30377/1859.44)	
**0.25**	**26h**	$2.0Gy - 3.3Gy^{(9)}$	$2.0Gy - 4.7Gy^{(10)}$	$3.10Gy^{(9)}/4.23Gy^{(10)}$	$4.7Gy$
		$[93Gy]^{\ddagger}$	$[126.9Gy]^{\ddagger}$	$[93Gy]^{\ddagger}/[126.9Gy]^{\ddagger}$	$[141Gy]^{\ddagger}$
		(192613/5840.14)		(331871/9068.78)/(52201/3183.69)	
	**18h**	$---$	$---$	$---$	$---$

Table S6: Performance of heterogeneous and homogeneous radiation therapies for the low migration case and fast-cycling CSCs. Data corresponding to 20 simulations (with different seeds of a random number generator) are presented. Treatment sessions were scheduled along 6 weeks (30 sessions) separated by 24 hours intervals except for weekends, where a 72 hours interval is allowed (†) and 7 days a week along 30 sessions at 24 hours intervals (without weekend interruptions) (‡). In the heterogeneous therapies, radiation doses are specified both for the outer (left) and inner (right) tumor regions, each case being indexed from (1) to (10). The averaged dose for any of the previous cases is labeled with the same number in the columns corresponding to homogeneous therapies. In brackets the total dose of the radiation therapy treatment and within braces the number of CSCs at the recurrence tumor stage with the corresponding standard deviation. See also Figure S5 where some of these results are represented.

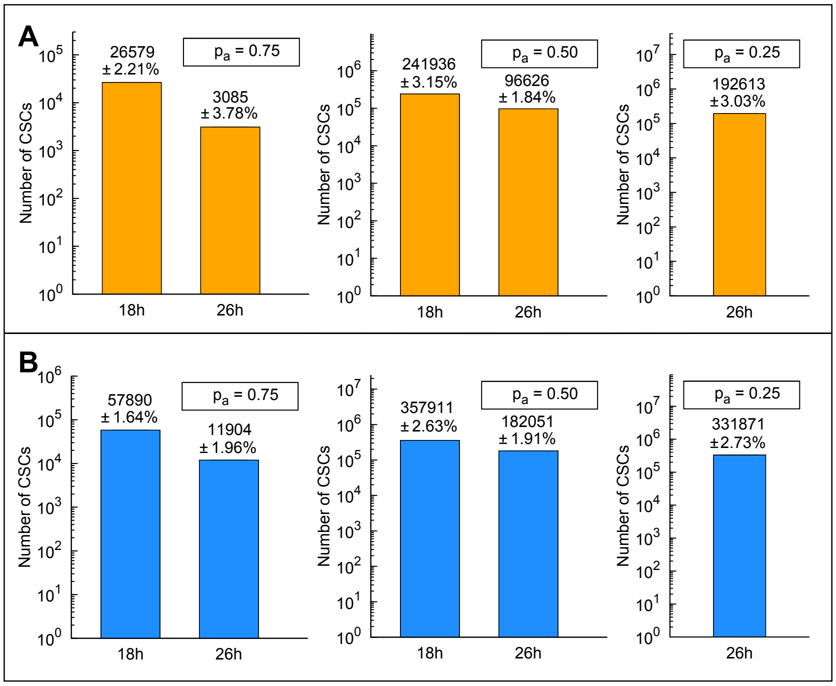

Figure S5: Total number of fast-cycling CSCs at the end of the recurrence tumor stage for heterogeneous and averaged homogeneous radiation therapies with the low migration rate. Number of CSCs at the end of the recurrence tumor stage (where about 10^6 cells are again present) and the corresponding standard deviations after performing 20 simulations in each case (with different seeds of a random number generator) are shown. (A) For heterogeneous therapies that do not achieve tumor control and (B) for the corresponding averaged homogeneous therapies considered in Table S6 for the cases $p_a = 0.75$, $p_a = 0.50$ and $p_a = 0.25$ (left, middle, right). The low migration rate and CSC cycle durations equal to $18h$ and $26h$ have been assumed. Notice that the vertical coordinate is represented in a logarithmic scale. See Table S6 for further details.

		Heterogeneous therapy		Homogeneous therapy	
p_a	τ_{csc}	**No Control**	**Control**	**No Control**	**Control**
**0.75**	**26h**	$2.0Gy - 2.5Gy^{(1)}$	$2.8Gy - 3.4Gy^{(2)}$	$2.14Gy^{(1)}/2.97Gy^{(2)}$	$3.4Gy$
		$[64.2Gy]^{\dagger}$	$[89.1Gy]^{\dagger}$	$[64.2Gy]^{\dagger}/[89.1Gy]^{\dagger}$	$[102Gy]^{\dagger}$
		(11050/484.08)		(18967/326.68)/(2269/189.34)	
	**18h**	$2.0Gy - 2.5Gy^{(3)}$	$3.6Gy - 4.3Gy^{(4)}$	$2.17Gy^{(3)}/3.83Gy^{(4)}$	$4.3Gy$
		$[65.1Gy]^{\dagger}$	$[114.9Gy]^{\dagger}$	$[65.1Gy]^{\dagger}/[114.9Gy]^{\dagger}$	$[129Gy]^{\dagger}$
		(159982/5495.70)		(197218/5508.36)/(3826/319.69)	
**0.50**	**26h**	$2.0Gy - 2.9Gy^{(5)}$	$3.5Gy - 4.1Gy^{(6)}$	$2.31Gy^{(5)}/3.70Gy^{(6)}$	$4.1Gy$
		$[69.3Gy]^{\ddagger}$	$[111Gy]^{\ddagger}$	$[69.3Gy]^{\ddagger}/[111Gy]^{\ddagger}$	$[123Gy]^{\ddagger}$
		(218770/5954.86)		(282412/6544.93)/(9844/562.32)	
	**18h**	$2.0Gy - 2.9Gy^{(7)}$	$4.4Gy - 4.9Gy^{(8)}$	$2.35Gy^{(7)}/4.60Gy^{(8)}$	$4.9Gy$
		$[70.5Gy]^{\ddagger}$	$[138Gy]^{\ddagger}$	$[70.5Gy]^{\ddagger}/[138Gy]^{\ddagger}$	$[147Gy]^{\ddagger}$
		(594483/8063.34)		(831328/18256.43)/(48205/2468.33)	
**0.25**	**26h**	$2.0Gy - 3.3Gy^{(9)}$	$4.3Gy - 4.8Gy^{(10)}$	$2.57Gy^{(9)}/4.52Gy^{(10)}$	$4.8Gy$
		$[77.1Gy]^{\ddagger}$	$[135.6Gy]^{\ddagger}$	$[77.1Gy]^{\ddagger}/[135.6Gy]^{\ddagger}$	$[144Gy]^{\ddagger}$
		(689546/11268.43)		(896248/19376.56)/(96035/4090.75)	
	**18h**	$---$	$---$	$---$	$---$

Table S7: Performance of heterogeneous and homogeneous radiation therapies for the high migration case and fast-cycling CSCs. Data corresponding to 20 simulations (with different seeds of a random number generator) are presented. Treatment sessions were scheduled along 6 weeks (30 sessions) separated by 24 hours intervals except for weekends, where a 72 hours interval is allowed (†) and 7 days a week along 30 sessions at 24 hours intervals (without weekend interruptions) (‡). In the heterogeneous therapies, radiation doses are specified both for the outer (left) and inner (right) tumor regions, each case being indexed from (1) to (10). The averaged dose for any of the previous cases is labeled with the same number in the columns corresponding to homogeneous therapies. In brackets the total dose of the radiation therapy treatment and within braces the number of CSCs at the recurrence tumor stage with the corresponding standard deviation. See also Figure S6 where some of these results are represented.

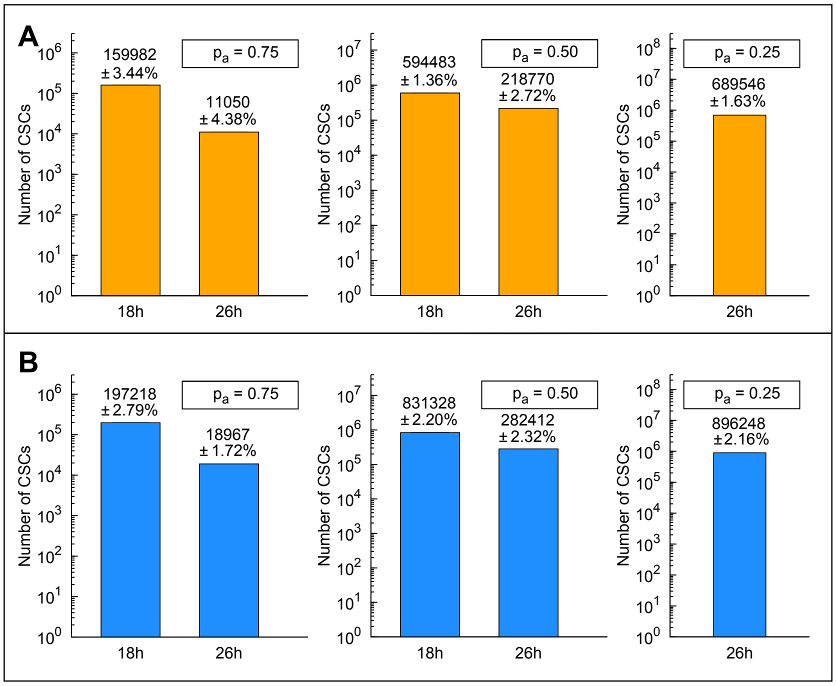

Figure S6: Total number of fast-cycling CSCs at the end of the recurrence tumor stage for heterogeneous and averaged homogeneous radiation therapies with the high migration rate. Number of CSCs at the end of the recurrence tumor stage (where about 10^6 cells are again present) and the corresponding standard deviations after performing 20 simulations in each case (with different seeds of a random number generator) are shown. (A) For heterogeneous therapies that do not achieve tumor control and (B) for the corresponding averaged homogeneous therapies considered in Table S7 for the cases $p_a = 0.75$, $p_a = 0.50$ and $p_a = 0.25$ (left, middle, right). The high migration rate and CSC cycle durations equal to $18h$ and $26h$ have been assumed. Notice that the vertical coordinate is represented in a logarithmic scale. See Table S7 for further details.

2.3.- A particular case of cancer cell plasticity.

To conclude this section, we describe an example of cancer cell plasticity, a hypothesis that has been proposed to better understand the onset of resistance after therapy (cf. for instance [30], [31] and [32]). To address this issue, we suppose that in addition to CSCs giving raise to CCs by asymmetric division, a (assumed small) percentage of CCs may transform to a CSC phenotype, possibly as a reaction to radiation therapy. In particular, we have considered the cases of CSC cycle duration equal to $48h$ and p_a equal to 0.75 and 0.25, both for the low and high migration cases respectively. We have then assumed that a small percentage of CCs (5%) are transformed to CSCs after sessions 5, 15 and 25 of a standard fractionation protocol (5 days a week along 30 sessions at 24 hours intervals except for weekends, where a 72 hours interval is allowed). As one can expect, any increase in the number of CSCs results in increased malignancy, measured in terms of higher radioresistance to therapy. However, our conclusion that heterogeneous, tumor-adapted therapies fare better than their corresponding averaged, homogeneous versions continues to hold. To show that, in Table S8 (respectively Table S9) a comparison is provided of the same cases with and without the cancer cell plasticity effect for the low (respectively high) migration rate. See also Figures S7 and S8 where some results provided in Tables S8 and S9 are shown respectively.

		Heterogeneous therapy		Homogeneous therapy	
p_a		**No Control**	**Control**	**No Control**	**Control**
**0.75**	**No CC Plasticity**	$---$	$2.0Gy - 2.7Gy^{(1)}$	$2.12Gy^{(1)}$	$2.7Gy$
			$[63.6Gy]$	$[63.6Gy]$	$[81Gy]$
				(514/13.65)	
	**CC Plasticity**	$2.0Gy - 2.7Gy^{(2)}$	$2.4Gy - 3.2Gy^{(3)}$	$2.12Gy^{(2)}/2.54Gy^{(3)}$	$3.2Gy$
		$[63.6Gy]$	$[76.2Gy]$	$[63.6Gy]/[76.2Gy]$	$[96Gy]$
		(771/24.37)		(1256/43.42)/(197/26.14)	
**0.25**	**No CC Plasticity**	$---$	$2.0Gy - 3.9Gy^{(4)}$	$2.47Gy^{(4)}$	$3.9Gy$
			[74.1Gy]	[74.1Gy]	[117Gy]
				(96346/1141.30)	
	**CC Plasticity**	$2.0Gy - 3.9Gy^{(5)}$	$3.2Gy - 4.3Gy^{(6)}$	$2.47Gy^{(5)}/3.47Gy^{(6)}$	$4.3Gy$
		$[74.1Gy]$	$[104.1Gy]$	$[74.1Gy]/[104.1Gy]$	$[129Gy]$
		(143242/4084.60)		(297794/6467.14)/(5144/280.49)	

Table S8: Estimating cancer cell plasticity effects for the low migration case and CSC cycle duration equal to 48 hours. In all cases, treatment sessions were scheduled along 6 weeks separated by 24 hours intervals except for weekends, where a 72 hours interval is allowed. Data corresponding to 20 simulations (with different seeds of a random number generator) are presented. In the heterogeneous therapies, radiation doses are specified both for the outer (left) and inner (right) tumor regions, each case being indexed from (1) to (6). The averaged dose for any of the previous cases is labeled with the same number in the columns corresponding to homogeneous therapies. In brackets the total dose of the radiation therapy treatment and within braces the number of CSCs at the recurrence tumor stage with the corresponding standard deviation. See also Figure S7 where some of these results are represented.

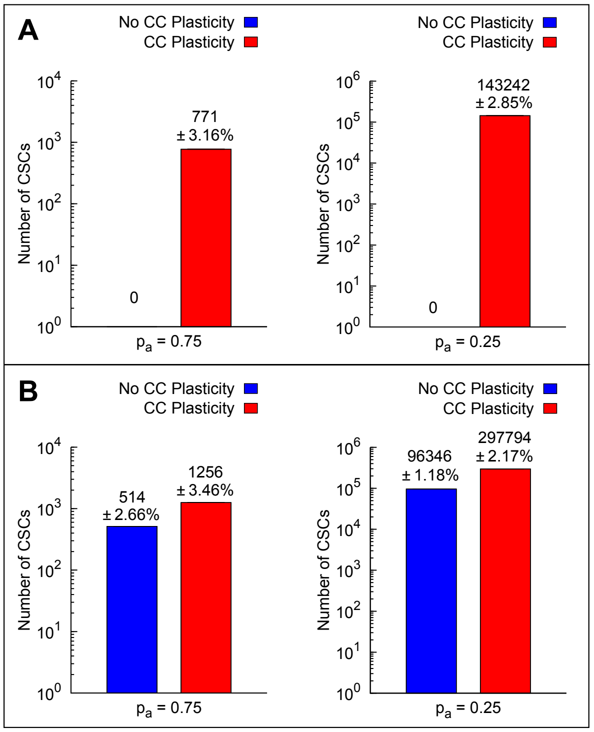

Figure S7: Cancer cell plasticity effects on the total number of CSCs at the end of the recurrence tumor stage for heterogeneous and averaged homogeneous radiation therapies. The low migration rate and CSC cycle duration equal to $48h$ have been assumed. Number of CSCs at the end of the recurrence tumor stage (where about 10^6 cells are again present) and the corresponding standard deviations after performing 20 simulations in each case (with different seeds of a random number generator) are shown. Results are provided for radiation therapies with and without cell plasticity effects. (A) For heterogeneous therapies that do not achieve tumor control and (B) for the corresponding averaged homogeneous therapies considered in Table S8 for the cases $p_a = 0.75$ and $p_a = 0.25$ (left and right). Notice that the vertical coordinate is represented in a logarithmic scale. See Table S8 for further details.

		Heterogeneous therapy		Homogeneous therapy	
p_a		**No Control**	**Control**	**No Control**	**Control**
**0.75**	**No CC Plasticity**	$---$	$2.2Gy - 2.7Gy^{(1)}$	$2.33Gy^{(1)}$	$2.7Gy$
			$[69.9Gy]$	$[69.9Gy]$	$[81Gy]$
				(267/10.61)	
	**CC Plasticity**	$2.2Gy - 2.7Gy^{(2)}$	$2.6Gy - 3.2Gy^{(3)}$	$2.33Gy^{(2)}/2.75Gy^{(3)}$	$3.2Gy$
		$[69.9Gy]$	$[82.5Gy]$	$[69.9Gy]/[82.5Gy]$	$[96Gy]$
		(953/37.45)		(2487/92.83)/(526/34.56)	
**0.25**	**No CC Plasticity**	$---$	$3.4Gy - 3.9Gy^{(4)}$	$3.60Gy^{(4)}$	$3.9Gy$
			$[108Gy]$	$[108Gy]$	$[117Gy]$
				(7716/174.66)	
	**CC Plasticity**	$3.4Gy - 3.9Gy^{(5)}$	$3.7Gy - 4.3Gy^{(6)}$	$3.60Gy^{(5)}/3.94Gy^{(6)}$	$4.3Gy$
		$[108Gy]$	$[118.2Gy]$	$[108Gy]/[118.2Gy]$	$[129Gy]$
		(8116/251.09)		(17212/592.21)/(3501/278.34)	

Table S9: Estimating cancer cell plasticity effects for the high migration case and CSC cycle duration equal to 48 hours. In all cases, treatment sessions were scheduled along 6 weeks separated by 24 hours intervals except for weekends, where a 72 hours interval is allowed. Data corresponding to 20 simulations (with different seeds of a random number generator) are presented. In the heterogeneous therapies, doses are specified both for the outer (left) and inner (right) tumor regions, each case being indexed from (1) to (6). The averaged dose for any of the previous cases is labeled with the same number in the columns corresponding to homogeneous therapies. In brackets the total dose of the radiation therapy treatment and within braces the number of CSCs at the recurrence tumor stage with the corresponding standard deviation. See also Figure S8 where some of these results are represented.

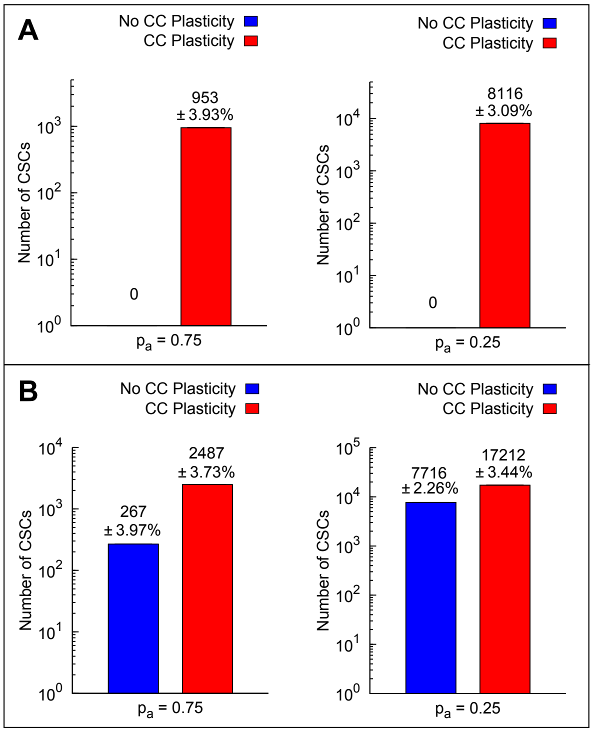

Figure S8: Cancer cell plasticity effects on the total number of CSCs at the end of the recurrence tumor stage for heterogeneous and averaged homogeneous radiation therapies. The high migration rate and CSC cycle duration equal to $48h$ have been assumed. Number of CSCs at the end of the recurrence tumor stage (where about 10^6 cells are again present) and the corresponding standard deviations after performing 20 simulations in each case (with different seeds of a random number generator) are shown. Results are provided for radiation therapies with and without cell plasticity effects. (A) For heterogeneous therapies that do not achieve tumor control and (B) for the corresponding averaged homogeneous therapies considered in Table S9 for the cases $p_a = 0.75$ and $p_a = 0.25$ (left and right). Notice that the vertical coordinate is represented in a logarithmic scale. See Table S9 for further details.

References

1. Drasdo D, Höhme S (2005) A single-cell-based model of tumor growth in vitro: monolayers and spheroids. Phys Biol 2: 133–47.

2. Radszuweit M, Block M, Hengstler JG, Schöll E, Drasdo D (2009) Comparing the growth kinetics of cell populations in two and three dimensions. Phys Rev E Stat Nonlin Soft Matter Phys 79.

3. Block M, Schöll E, Drasdo D (2007) Classifying the growth kinetics and surface dynamics in growing cell populations. Phys Rev Lett 99: 248101–104.

4. Honerkamp J (1993) In: Stochastic Dynamical Systems, Wiley-VCH.

5. Drasdo D (2005) Coarse graining in simulated cell populations. Advances in Complex Systems (ACS) 8: 319–63.

6. Gao X, McDonald JT, Hlatky L, Enderling H (2013) Acute and fractionated irradiation differentially modulate glioma stem cell division kinetics. Cancer Res 73: 1481–90.

7. Fowler JF (1989) The linear-quadratic formula and progress in fractionated radiotherapy. Br J Radiol 62: 679–94.

8. Lee SP, Leu MY, Smathers JB, McBride WH, Parker RG, et al. (1995) Biologically effective dose distribution based on the linear quadratic model and its clinical relevance. Int J Radiat Oncol Biol Phys 33: 375–89.

9. Cruchten SV, Broeck WVD (2002) Morphological and biochemical aspects of apoptosis, oncosis and necrosis. Anat Histol Embryol 31: 214–23.

10. Bursch W, Kleine L, Tenniswood M (1990) The biochemistry of cell death by apoptosis. Biochem Cell Biol 68: 1071–4.

11. Goergen JL, Marc A, Engasser JM (1993) Determination of cell lysis and death kinetics in continuous hybridoma cultures from the measurement of lactate dehydrogenase release. Cytotechnology 11: 189–95.

12. Wells JE, Russell JB (1996) The effect of growth and starvation on the lysis of the ruminal cellulolytic bacterium fibrobacter succinogenes. Appl Environ Microbiol 62: 1342–6.

13. Enderling H, Park D, Hlatky L, Hahnfeldt P (2009) The importance of spatial distribution of stemness and proliferation state in determining tumor radioresponse. Math Model Nat Phenom 4: 117–33.

14. Allam A, Taghian A, Gioioso D, Duffy M, Suit HD (1993) Intratumoral heterogeneity of malignant gliomas measured in vitro. Int J Radiat Oncol Biol Phys 27: 303–8.

15. Allalunis-Turner MJ, Barron GM, 3rd Day RS, Fulton DS, Urtasun RC (1992) Radiosensitivity testing of human primary brain tumor specimens. Int J Radiat Oncol Biol Phys 23: 339–43.

16. Taghian A, Ramsay J, Allalunis-Turner MJ, Budach W, Gioioso D, et al. (1993) Intrinsic radiation sensitivity may not be the major determinant of the poor clinical outcome of glioblastoma multiforme. Int J Radiat Oncol Biol Phys 25: 243–9.

17. Dionysiou DD, Stamatakos GS, Gintides D, Uzunoglu N, Kyriaki K (2008) Critical parameters determining standard radiotherapy treatment outcome for glioblastoma multiforme: a computer simulation. Open Biomed Eng J 2: 43–51.

18. Enderling H, Hlatky L, Hahnfeldt P (2013) Cancer stem cells: A minor cancer subpopulation that redefines global cancer features. Front Oncol 3.

19. Gillespie DT (1977) Exact stochastic simulation of coupled chemical reactions. J Phys Chem 81: 2340–61.

20. Clarke MF, Dick JE, Dirks PB, Eaves CJ, Jamieson CH, et al. (2006) Cancer stem cells–perspectives on current status and future directions: Aacr workshop on cancer stem cells. Cancer Res 66: 9339–44.

21. Bonnet D, Dick JE (1997) Human acute myeloid leukemia is organized as a hierarchy that originates from a primitive hematopoietic cell. Nat Med 3: 730–7.

22. Li L, Neaves WB (2006) Normal stem cells and cancer stem cells: the niche matters. Cancer Res 66: 4553–57.

23. Stupp R, Hegi ME (2007) Targeting brain-tumor stem cells. Nat Biotechnol 25: 193–4.

24. Dembinski JL, Krauss S (2009) Characterization and functional analysis of a slow cycling stem cell-like subpopulation in pancreas adenocarcinoma. Clin Exp Metastasis 26: 611–23.

25. Roesch A, Fukunaga-Kalabis M, Schmidt EC, Zabierowski SE, Brafford PA, et al. (2010) A temporarily distinct subpopulation of slow-cycling melanoma cells is required for continuous tumor growth. Cell 141: 583–94.

26. Moore N, Houghton J, Lyle S (2012) Slow-cycling therapy-resistant cancer cells. Stem Cells Dev 21: 1822–30.

27. Moore N, Lyle S (2011) Quiescent, slow-cycling stem cell populations in cancer: a review of the evidence and discussion of significance. J Oncol 2011.

28. Richichi C, Brescia P, Alberizzi V, Fornasari L, Pelicci G (2013) Marker-independent method for isolating slow-dividing cancer stem cells in human glioblastoma. Neoplasia 15: 840–7.

29. Bansal A, Ramalingam S, Anant S (2013) Cancer stem cells in the origin and transformation of barrett's esophagus: Current knowledge and areas of uncertainty. Immunogastroenterology 2: 9–21.

30. Chaffer CL, Brueckmann I, Scheel C, Kaestli AJ, Wiggins PA, et al. (2011) Normal and neoplastic nonstem cells can spontaneously convert to a stem-like state. Proc Natl Acad Sci U S A 108: 7950–5.

31. Tang DG (2012) Understanding cancer stem cell heterogeneity and plasticity. Cell Res 22: 457–72.

32. Meacham CE, Morrison SJ (2013) Tumour heterogeneity and cancer cell plasticity. Nature 501: 328–37.
